# The presence of plasmids in bacterial hosts alters phage isolation and infectivity

**DOI:** 10.1038/s43705-022-00158-9

**Published:** 2022-08-19

**Authors:** Lyman Ngiam, Karen D. Weynberg, Jianhua Guo

**Affiliations:** 1 Australian Centre for Water and Environmental Biotechnology, The University of Queensland, Brisbane, QLD 4072 Australia; 2 Australian Centre for Ecogenomics, School of Chemistry and Molecular Biosciences, The University of Queensland, Brisbane, QLD 4072 Australia

**Keywords:** Bacteriophages, Virus-host interactions

## Abstract

Antibiotic resistance genes are often carried by plasmids, which spread intra- and inter genera bacterial populations, and also play a critical role in bacteria conferring phage resistance. However, it remains unknown about the influence of plasmids present in bacterial hosts on phage isolation and subsequent infectivity. In this study, using both *Escherichia coli* and *Pseudomonas putida* bacteria containing different plasmids, eight phages were isolated and characterized in terms of phage morphology and host range analysis, in conjunction with DNA and protein sequencing. We found that plasmids can influence both the phage isolation process and phage infectivity. In particular, the isolated phages exhibited different phage plaquing infectivity towards the same bacterial species containing different plasmids. Furthermore, the presence of plasmids was found to alter the expression of bacteria membrane protein, which correlates with bacterial cell surface receptors recognized by phages, thus affecting phage isolation and infectivity. Given the diverse and ubiquitous nature of plasmids, our findings highlight the need to consider plasmids as factors that can influence both phage isolation and infectivity.

## Introduction

Bacteriophages or phages, are viruses that specifically infect and kill bacteria. With a total estimated global abundance of 10^31^ particles, phages represent the most abundant entities found on Earth and can be found in every living habitat where bacteria reside. Given their vast abundance and diversity, phages also play an important role in regulating different populations within bacterial communities in both natural and engineered ecosystems [1]. Owing to the intrinsic properties of phage capable of infecting and killing bacteria, increasing studies have been utilizing phages as alternative antimicrobial agents for treatment of bacterial infections, also known as phage therapy. The surging interest in phage therapy observed globally coincides with the rise of antibiotic resistance, a major health crisis caused by abuse and misuse of antibiotics [2, 3]. Indeed, previous studies have shown that phage therapy offers promising antimicrobial efficiencies with several added advantages compared to antibiotics [1, 4]. Despite these advantages, phage therapy also has certain limitations, including host range specificity and development of phage resistance [5, 6].

The development of phage resistance by bacteria represents an intrinsic defence mechanism against phages. Common phage resistance mechanisms include blocking of phage adsorption entry, interference of phage genome entry and replication [7, 8]. In addition, plasmids present in bacterial hosts are also found to be able to mediate the development of phage resistance [9–11]. Independent of bacterial chromosomal DNA, plasmids are circular, extrachromosomal DNA molecules that can encode accessory genes that confer selective advantage for bacterial survival such as antibiotic resistance, production of toxins as well as utilization of specific substrates for growth [12, 13]. Plasmid-mediated phage defence that leads to phage abortive infection systems was reported to be linked to the presence of a plasmid encoded toxin-antitoxin pair system, which is able to interfere with phage replication through reduced cell metabolism [14] or active promotion of programmed cell death [15, 16]. Apart from the known anti-phage immunity associated with plasmids, previous studies have also reported the association of plasmid and its encoded plasmid sex apparatus (i.e. pilus) that led to isolation of plasmid dependent phage such as *E. coli Inoviruses* targeting the F sex pilus [17] and broad host-range *Tectiviridae*-like PRD1 targeting conjugative pili of plasmids [18]. Despite the known role of plasmid-mediated phage defence and plasmid encoded sex-apparatus, information with regards to interactions of plasmids towards phage isolation and infectivity is scarce. Given the surging interest in therapeutic use of phage as alternative antimicrobial agents, there is a knowledge gap in investigating the potential roles of plasmids towards different phage aspects, such as isolation and infectivity. The objective of this study was to investigate whether and how the presence of plasmids in bacterial hosts could affect phage isolation and infectivity. To address this, *Escherichia coli* (*E. coli*) and *Pseudomonas putida* (*P. putida*) bacteria were selected and modified here to contain different type of conjugative plasmids that belong to different incompatibility groups (including lncP-1 α, IncP-1 ε and lncA/C2 respectively). Subsequently, phage isolations were individually performed, and phage isolates were subjected to further phage characterization in terms of phage morphology, adsorption, one-step phage growth curves, phage host range analysis and efficiency of plating. Additionally, bacterial total protein profiling, as well as genomic screening of phage-resistant mutants, were also performed to assess the abundance of bacterial membrane protein and its correlation to phage receptors identified. Our findings offer insights into the roles of plasmids harboured in bacterial hosts in the phage isolation process and subsequently infectivity of phage.

## Materials and methods

### Bacterial strains and culture conditions

The bacterial strains used are listed in Table 1. In this study, *E. coli* K12 and *P. putida* KT2440 lab strain model bacteria were selected to investigate the effect of plasmids toward phage isolation and infectivity. Additionally, the two different bacteria genera were selected to eliminate possible genus-specific phenomena. For the two groups of bacteria, each group of bacteria harbouring three different conjugative plasmids. Plasmid RP4 [19] and pKJK5 [20] are conjugative multi-drug resistant plasmids that belong to incompatibility group P-1 (type α and ε respectively) whereas plasmid pMS6198A is a conjugative multi-drug resistant plasmid that belongs to incompatibility group A/C2 [21]. A control strain containing no plasmid was also included in each group of bacteria. All bacterial strains were cultivated in Luria-Bertani (LB) liquid broth or LB with 1% agar solid medium and incubated at 30 °C (*P. putida*) or 37 °C (*E. coli*). When required, ampicillin (100 µg/mL), tetracycline (20 µg/mL) or kanamycin (100 µg/mL) was added to maintain plasmid presence in bacteria. Full details for plasmids are described in Table S1.

**Table 1:** Summary detail of the bacteria strains, plasmids and the phages isolated in this study.

Bacteria	Plasmid	Plasmid description	Phage isolated in this study	Taxonomy classification of phage (Family level)	Phage adsorption assay		Phage one step growth curve	
					Time needed (mins) to achieve 90% adsorption	Adsorption constant (ml/min)	Latent period (mins)	Burst size
*E. coli* K12 MG1655 wild type	N/A	N/A	vB_EcoM_LNA1 (herein refer as A1)	*Myoviridae*	20	1 × 10^−6^ ± 8 × 10^−8^	15	134 ± 4
*E. coli* K12 MG1655 with RP4	RP4	lncP-1 α; conjugative	vB_EcoM_LNA2 (herein refer as A2)	*Myoviridae*	20	7.7 × 10^−7^ ± 3 × 10^−8^	10	68 ± 2
*E. coli* K12 MG1655 with pMS6198A	pMS6198A	lncA/C2; conjugative	vB_EcoM_LNA6 (herein refer as A6)	*Myoviridae*	12	3.8 × 10^−7^ ± 1.1 × 10^−8^	20	80 ± 5
*E. coli* K12 MG1655 with pKJK5	pKJK5	lncP-1 ε; conjugative	vB_EcoP_LNA7 (herein refer as A7)	*Autographiviridae*	10	2.9 × 10^−7^ ± 1.6 × 10^−8^	15	18 ± 4
*P. putida* KT2440 wild type	N/A	N/A	vB_PputP_LNA8 (herein refer as A8)	*Autographiviridae*	40	1.6 × 10^−6^ ± 8.5 × 10^−8^	10	6 ± 2
*P. putida* KT2440 with RP4	RP4	lncP-1 α; conjugative	vB_PputP_LNA9 (herein refer as A9)	*Tectiviridae*	60	2 × 10^−7^ ± 2.1 × 10^−8^	10	69 ± 4
*P. putida* KT2440 with pMS6198A	pMS6198A	lncA/C2; conjugative	vB_PputP_LNA10 (herein refer as A10)	*Autographiviridae*	15	1.1 × 10^−6^ ± 9.5 × 10^−8^	30	6 ± 1
*P. putida* KT2440 with pKJK5	pKJK5	lncP-1 ε; conjugative	vB_PputP_LNA11 (herein refer as A11)	*Autographiviridae*	35	8.4 × 10^−7^ ± 5.5 × 10^−7^	10	38 ± 5

Details for isolated phage taxonomic classification at family level and its population dynamic is also listed in this table.

### Bacteriophage isolation, purification and propagation

Using the individual bacterial hosts listed in Table 1, bacteriophages were isolated using domestic wastewater collected from a full-scale wastewater treatment plant (WWTP) in Brisbane, Australia. The procedure for phage isolations and purifications were performed as previously described [22] with slight modifications. Temperature incubation for *P. putida* bacteria and *E. coli* bacteria were performed at 30 °C and 37 °C, respectively. The isolated, purified phage isolates were propagated by infecting bacteria host at mid-exponential growing phase (OD_600_ = 0.3, ~1 × 10^8^ CFU/mL) in LB liquid medium and incubated at 30 °C (for *P. putida* bacteria) or 37 °C (for *E. coli* bacteria) until clear lysates occurred. Next, phage lysates were collected via centrifugation (12,000 × *g*, 10 min, 4 °C), followed by filtration with 0.22 µm PES filter membrane. Additionally, plaque assays were performed to assess titre of phages post-propagation.

### Phage host range analysis and efficiency of plating (EOP)

Phage host range spectrum was determined using spot lysis assay as previously described [22]. The host range spectrum of phage was determined according to morphology of the spot plaque formed, which can be classified into three groups: (++) clear spot plaques, (+) turbid spot plaques, and (−) no plaques. To further validate the observed phage host range via spot lysis assay, an EOP assay was also conducted according to procedure previously described [23]. The EOP of phage was calculated by dividing the average phage titre on permissive bacteria host over phage titre on original host bacteria (i.e. bacterial host where phage is isolated from) which has an EOP value of 1.

### Phage adsorption assay

The determination of phage adsorption was carried out as previously described [24] with some modifications. Briefly, purified phage lysates were added at multiplicity of infection (MOI) of 0.1 to bacteria host grown at mid-exponential phase (OD_600_ = 0.3, ~1 × 10^8^ CFU/mL) in a 20 mL LB medium. Upon phage inoculation, 1 mL of phage-host suspensions were immediately aliquoted for determination of phage titre. This also marks the phage titre at time point (t = 0). Thereafter, same volume of phage-host suspensions were sampled every 4 min, for a total period of 20 min. For phage displaying prolonged adsorption, the phage host suspensions were sampled every 10 mins after 20 min, for a total period of 80 min. Finally, the phage titres result expressed in plaque forming units/mL (PFU/mL) were plotted against time to determine phage adsorption rate.

### One-step phage growth curve

One-step growth curve experiments were conducted as described previously [24] with slight modifications. Briefly, purified phage lysates were added at MOI ratio of 0.1 to bacterial host culture isolates grown at mid-exponential phase (OD_600_ = 0.3, ~1 × 10^8^ CFU/mL) and allowed for phage adsorption according to the phage adsorption assay determined previously. After phage adsorption, the phage-host suspensions were centrifuged (6,000 × *g*, 10 min, 25 °C) to remove any unabsorbed free phage. After centrifugation, the supernatant was discarded and the pellet was resuspended in 30 mL of fresh LB medium, and incubated with shaking at 30 °C (*P. putida* strains) or 37 °C (*E. coli* strains). Prior to incubation, an aliquot of 1 mL was immediately collected for determination of phage titre at time point (t = 0). Next, the same volume of aliquot was sampled every 5 min for the first 20 min, and thereafter every 10 min for a total period of 80 min. The phage titres at different time points expressed in PFU/mL were then plotted against time to determine phage latent period and burst size. The burst size of phage was calculated by dividing the phage titres at plateau phase by the initial number of infected bacterial cells (~1 × 10^8^ CFU/mL).

### Transmission electron microscopy (TEM)

TEM was performed to visualize the morphology of the phage isolated. Using the purified phage lysates, an aliquot of phage lysate (2 µL) was dropped onto a 200-mesh carbon coated copper grid and negatively stained with 1% (wt/vol) uranyl acetate and air dried. Next, the grid was examined using a transmission electron microscope (JEM-1011, JEOL Ltd. Tokyo, Japan) with accelerating voltage of 80 kV. TEM images were analysed using ImageJ [25].

### *E. coli* bacteria total protein extraction and proteomic analysis

Total bacterial protein of *E. coli* bacteria containing different plasmids were extracted and compared against wild type *E. coli* containing no plasmid. From the total bacteria protein analyzed, bacterial membrane protein that were previously identified as potential cell surface receptor for phages are selected for further analysis [26, 27]. Details for protein extraction and proteomic analysis are described in Text S1.

### Phage DNA extraction and genome sequencing

The extraction of phage DNA was carried out using a phage DNA isolation kit (Norgen Biotek corp., Thorold, Canada) according to manufacturer’s protocol. Both Qubit fluorometer (Thermo Fisher Scientific) and Nanodrop (Nanodrop Technologies, USA) were used to assess the quantity and quality of phage DNA prior to sequencing. Next, the extracted phage DNA were submitted to the Australian Centre for Ecogenomics for whole genome sequencing using NextSeq (Illumina) genome sequencing (1Gbp sequencing depth) as well as Nanopore sequencing (48 hour run). Sequence data returned in raw FASTQ format (Illumina) and FAST5 format (Nanopore) were quality checked, trimmed, and hybrid assembled using Unicycler v0.4.9 [28]. Full details for processing raw Illumina and Nanopore sequencing data prior to assembly are described in Text S2.

### Bioinformatic analysis of phage genome sequence

The resulting hybrid assembled phage complete draft genomes from Unicycler were annotated using various annotation programs such as PHANOTATE [29], GeneMark [30] and Glimmer [31]. The returned results of predicted ORFs were functionally annotated against the non-redundant protein sequence database using the BLAST tools at NCBI (http://blast.ncbi.nlm.nih.gov/Blast.cgi) [32]. Putative tRNAs within the phage genome were predicted using tRNA Scan-SE (http://lowelab.ucsc.edu/tRNAscan-SE) tool. Prediction of the phage lifestyle was performed using Phage Classification Tool Set (PHACTS) [33]. The presence of antibiotic resistance and virulence factor associated genes within phage genomes was analyzed using ResFinder and VirulenceFinder, web-based service tools that are available at Centre for Genomic Epidemiology (http://www.genomicepidemiology.org/). Genome comparison between the isolated phage and were visualized using BLAST Ring Image Generator (BRIG) [34] and Easyfig [35]. Phylogenetic analysis of the isolated phages were compared using phage terminase large subunit amino acid sequences. The amino acid sequences were aligned using ClustalW algorithm [36] and a maximum-likelihood tree was constructed using MEGA X (http://www.megasoftware.net/).

### Phage resistant mutant sequencing

Phage resistant bacterial mutants were generated by infecting the bacterial host to single phage treatment. Briefly, to a 3 ml LB agarose, 300 µl of fresh overnight bacteria culture (approximate 1 × 10^9^ CFU/mL) inoculum and 100 µl of serially diluted phage (up to 10^-6^) were added and plated on a LB agar plate. Next, the plates were incubated at 37 °C overnight. Bacterial colonies that were able to grow in the presence of infectious phage in LB agar plates were isolated and verified via spot assay to be phage resistant mutant strains. Three phage resistant mutant colonies generated per phage type were selected for subsequent DNA extraction. All phage-resistant mutants strains generated from each individual phage treatment were subsequently grown in LB broth overnight and subjected to bacterial whole genome DNA extraction. DNA of both phage sensitive and phage resistant mutant strains were extracted using GenElute™ Bacterial Genomic DNA Kit (Sigma-Aldrich, Australia) according to manufacturer’s protocol. The final DNA extracted was submitted to Novogene facilities (Singapore) for Illumina sequencing service with sequencing output of 1Gb per samples. Raw reads generated from the sequencing were paired end, quality checked and trimmed using Geneious Prime 2020.0.5 (https://www.geneious.com). Next, the processed reads were assembled by mapping against closely related reference genomes available at NCBI database (*E. coli* K12 MG1655 U00096.2) using Geneious plugins BowTie2 tool [37]. Single nucleotide polymorphisms and indel variants were performed using Geneious plugins FreeBayes tool [38].

### Statistical analysis

All experiments were done in triplicate, and the values were reported in the format of mean ± SD. One-way ANOVA using multiple Dunnett’s test was used for determination of statistical significance using Graph Pad Prism 6.05 software. *P* value < 0.05 was considered as statistically significant.

## Results

### Phage isolation and morphology characterization

**Fig. 1:**
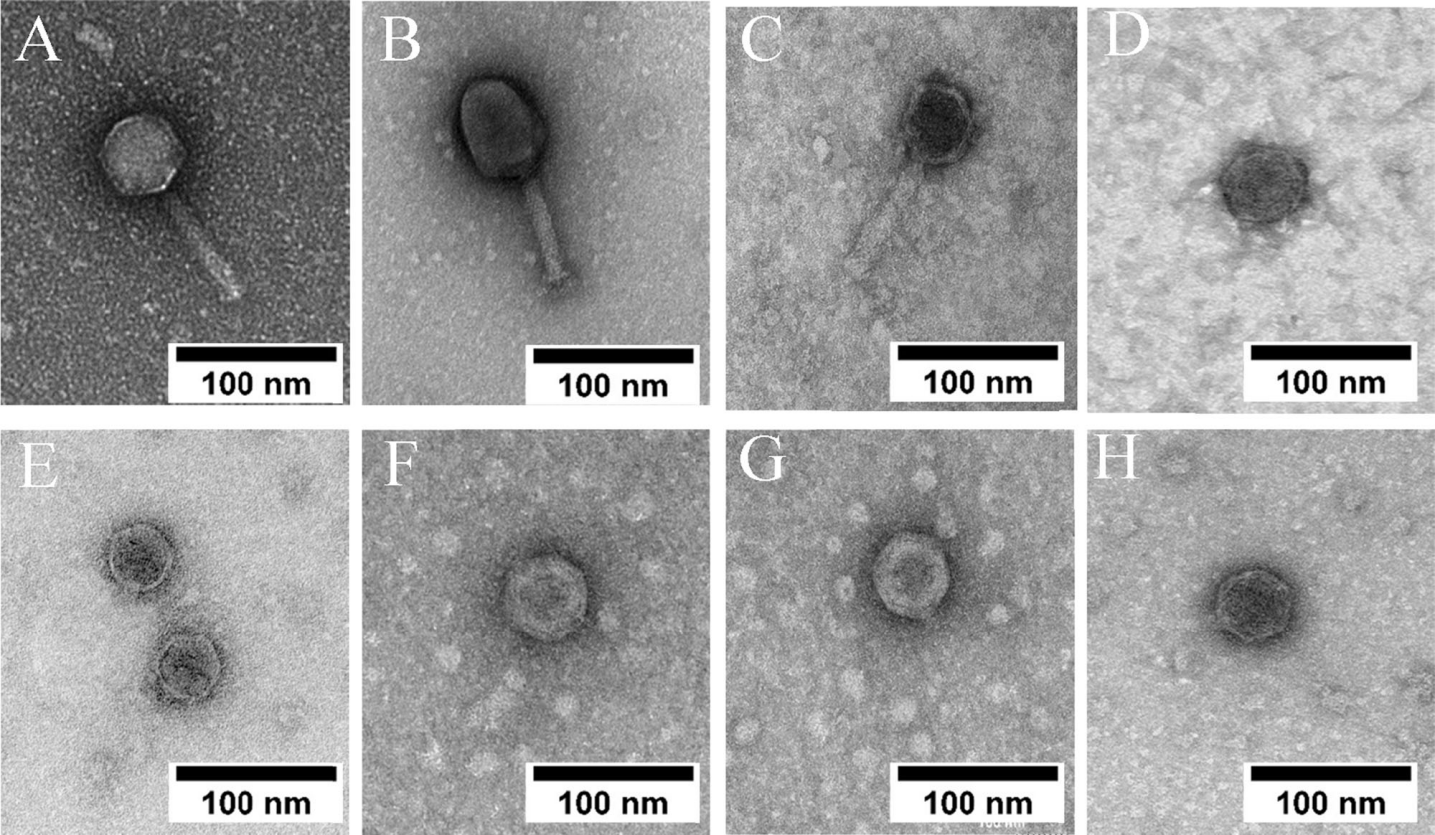
Morphology of phage isolates examined via TEM. TEM images of *E. coli* K12 phages represented in the top panel (**A**) phage A1 (**B**) phage A2 (**C**) phage A6 and (**D**) phage A7. *P. putida* phages represented in the bottom panel (**E**) phage A8 (**F**) phage A9 (**G**) phage A10 and (**H**) phage A11.

A total of 8 different phages were isolated using bacterial strains as hosts listed in Table 1. Phages A1 and A2 were isolated in a previous study [22] using the same wastewater source, whereas all other phages were isolated in this study. Each phage isolated was subsequently assigned a unique identifier according to the guideline recommended by the International Committee on Taxonomy of Viruses (ICTV). TEM was performed to visualize the morphology of the isolated phages (Fig. 1). All phages were found displaying morphologies that resemble different tail structured phages. *E. coli* K12 phage A1, A2 and A6 were found to adopt morphology similar to *Myoviridae*-like tailed phage with an average measured size range between 200 and 220 nm while phage A7 was found to adopt morphology similar to *Podoviridae-*like tailed phage. All of *P. putida* KT2440 phages (phage A8, A9, A10 and A11) were found to have morphology similar to *Podoviridae* tailed phage, with average measured size ranging between 55 and 60 nm.

### Phage host range determination and efficiency of plating (EOP)

Spot test assays were conducted to determine the host range of each phage isolated (Table S13). Phages isolated from the *E. coli* K12 bacteria group, were able to produce clear lysis spots in all of the *E. coli* K12 bacteria, except for phage A7. For phage A7, clear lysis spot was observed in *E. coli* K12 harbouring plasmid pMS6198A and pKJK5. For the other *E. coli* bacteria (wild type *E. coli* K12 and *E. coli* K12 harbouring plasmid RP4), turbid lysis spots were observed after spotting phage A7 on the bacterial lawn (Fig. S15A). Phages isolated from *P. putida* KT2440, namely phages A8, A10 and A11 were found able to infect all of the *P. putida* KT2440 strains while phage A9 was found able to produce clear spots in both *P. putida* KT2440 as well as *E. coli* K12 when harbouring plasmids RP4 and pKJK5.

**Fig. 2:**
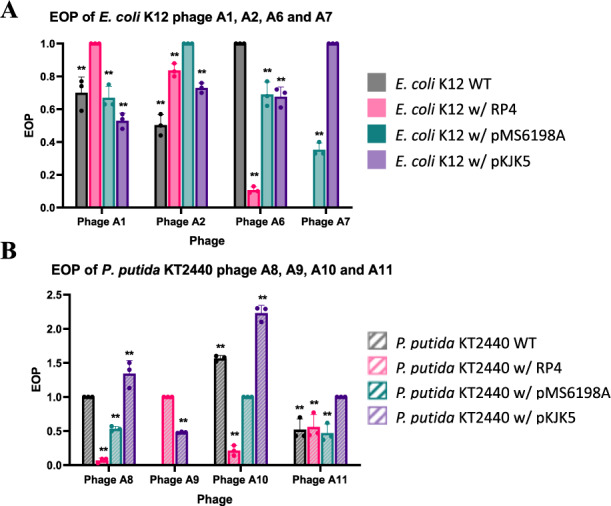
EOP of phages conducted against its original bacteria host and other permissive bacteria host. **A** EOP of *E. coli* K12 phages (**B**) EOP of *P. putida* KT2440 phages.

EOP test assay was performed to further validate the infectivity of phage isolates observed during the spot test assay. The EOP of phage isolates were compared between its original host (EOP = 1) and other permissive hosts. From the result, the EOP of all *E. coli* K12 phages (Fig. 2A) were found to have a significantly lower EOP value (*p* < 0.05) in other permissive *E. coli* K12 bacteria hosts when compared to its original host (Table S1). The EOP of *E. coli* phage A7 was not determined against wild type *E. coli* K12 and *E. coli* K12 harbouring RP4 plasmid as plaque formations were not observed. The EOP of *P. putida* KT2440 phages (Fig. 2B) were found also found to exhibit a lower EOP value (*p* < 0.05) in all other permissive *P. putida* KT2440 bacteria hosts except for phage A8 and A10. Both phage A8 and A10 were found to exhibit a high EOP value in some other *P. putida* KT2440 permissive bacteria hosts when compared to its original bacteria host. Specifically, phage A8 exhibits a high EOP value in *P. putida* KT2440 bacteria harbouring plasmid pKJK5 (EOP = 1.34 ± 0.19, *p* < 0.05) whereas phage A10 shows a high EOP value in wild-type *P. putida* KT2440 bacteria (EOP = 1.57 ± 0.04, *p* < 0.05) and *P. putida* KT2440 bacteria harbouring with pKJK5 plasmid (EOP = 1.57 ± 0.04, *p* < 0.05). Meanwhile, phage A9 exhibits a low EOP value (*p* < 0.05) against all other permissive *E. coli* K12 and *P. putida* KT2440 bacteria hosts.

### Phage latency period and burst size determination

The population dynamics of each phage isolated was determined by infecting the phage against the original bacterial strain used for its isolation in terms of adsorption assay and one-step growth curves (Table 1). Both *E. coli* K12 and *P. putida* KT2440 phages were found to have a range of adsorption kinetics ranging from 10 mins to 60 mins. *P. putida* KT2440 phage A9 has the slowest adsorption kinetics, where more than 90% phage adsorption is achieved after 60 mins. Meanwhile, *E. coli* K12 phage A7 displayed the fastest adsorption kinetics, with more than 90% phage adsorption achieved within 10 min. The one-step growth curve of phage isolated from this study also exhibited clear differences. For example, *P. putida* KT2440 phage A10 exhibited the longest latent period of 30 min, while all other phage isolates were found to have average latent periods ranging between 10–20 min.

Within the context of burst size, all *E. coli* K12 phage isolates except phage A7 were found to have an average burst size above 50, with phage A1 demonstrating the highest burst size (134 ± 4). The average burst size observed in *P. putida* KT2440 phages also varies. Specifically, phage A9 has the highest average burst size followed by phage A11. Both phage A8 and A10 were found to have the lowest burst size and have similar average burst size of 6.

### Genomic comparison of phages

Genomic DNA for phages were extracted to assess and compare the genomic traits of the phages isolated in this study. The genomic features of phage isolated are summarized in Table 2 (Full graphical presentation of phage genome features are presented in Supplementary Figs. S1–S8). From the results, we can further confirm that different phages were isolated despite the using the same host bacterium, depending on the specific plasmid presented in the host. Specifically, the genome size for *E. coli* K12 phages (phages A1, A2, A6 and A7 respectively) varied from 39,592 bp to 168,414 bp, with GC content ranged between 37 to 48.6%. Amongst the ORFs identified, 38 ORFs in phage A1, 119 ORFs in phage A2, 53 ORFs in phage A6, and 42 ORFs in phage A7 were annotated as putative functional phage proteins. The genomic features of *P. putida* KT2440 phages (phage A8, A9, A10 and A11 respectively) varied from 14,342 bp to 40,331 bp, with GC content ranged between 48.3 to 61.4%. Additionally, 36 ORFs in phage A8, 31 ORFs in phage A9, 18 ORFs in phage A10 and 23 ORFs in phage A11 were annotated as putative functional phage proteins. The remaining ORFs with unknown function in both *E. coli* K12 and *P. putida* KT2440 phages were annotated as hypothetical proteins.

**Table 2:** Summary of genomic features of *E. coli* K12 and *P. putida* KT2440 phages.

	Phage	Total size (bp)	GC content %	Number of ORFs	Number of ORFs with putative function	Number of tRNAs
*E. coli* K12 phages	vB_EcoM_LNA1	88,369	39.1	126	38	16
	vB_EcoM_LNA2	168,414	37.7	267	119	2
	vB_EcoM_LNA6	88,347	38.9	154	53	23
	vB_EcoP_LNA7	39,592	48.6	60	42	NA
*P. putida* KT2440 phages	vB_PputP_LNA8	40,103	61.4	54	36	NA
	vB_PputP_LNA9	14,342	48.3	43	31	NA
	vB_PputP_LNA10	39,974	61.0	50	18	NA
	vB_PputP_LNA11	40,331	57.7	56	23	NA

**Fig. 3:**
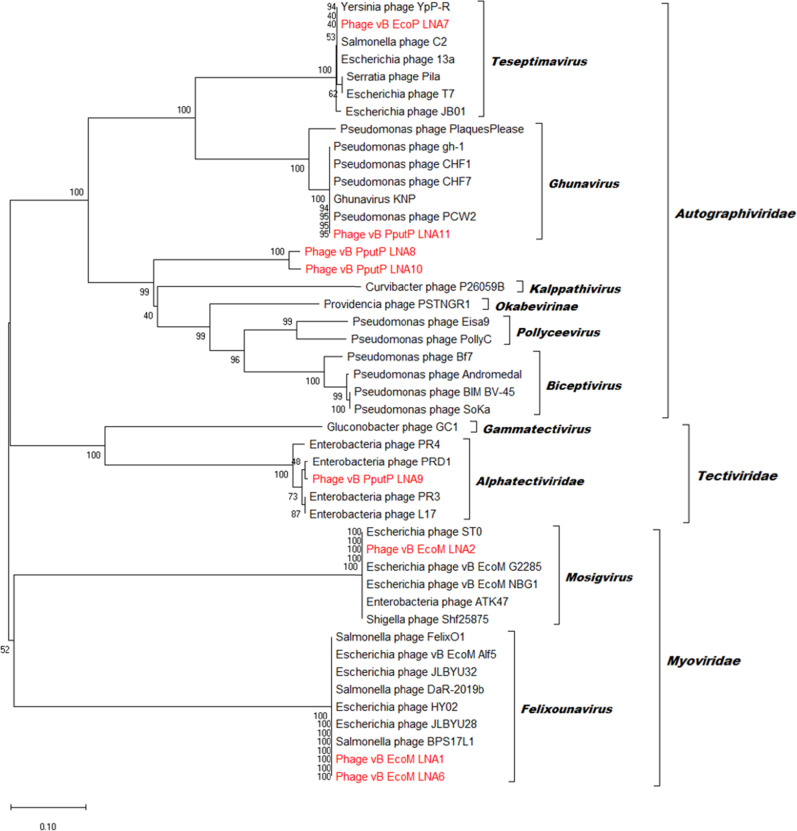
Phage proteomic phylogenetic tree constructed using phage-encoded protein terminase large subunit. Phages isolated in this study is highlighted in red. The phylogenetic tree was generated using the neighbour joining method and 1000 bootstrap replicates. The number at each node represents the bootstrap values based on 1000 resampling. Scale bar indicates 0.2 amino acid substitution per residue. The taxonomic label of the phylogenetic tree was labelled (from left to right) at genus level classification and family level classification.

Amongst assembled genomes of both *E. coli* K12 and *P. putida* KT2440 phages, phage A6 were found to have the highest number of tRNAs detected, followed by phages A1 and A2. For all other phage isolates, no tRNA genes were detected. Further genomic analyses using PHACTS, ResFinder and VirulenceFinder tools were used to predict the lifestyle of phage isolated in this study, as well as detection of antibiotic resistance or virulence associated genes within phage genomes. PHACTS analysis predicted lytic lifestyle for all phages except for phage A8, which were predicted to be non-confidently temperate lifestyle (Table S3). Meanwhile, no antibiotic resistance or virulence associated genes were detected within the genomes of any of the phage isolates. Whole genome comparison was performed to distinguish the relatedness of *E. coli* K12 and *P. putida* KT2440 phages isolated (Fig. S13). For the *E. coli* K12 phages isolated (Fig. S13A), phage A6 was observed to share high similarity to phage A1 (coverage: 92%; identity: 97%) but little or low similarity to other phages. For *P. putida* KT2440 phages isolated (Fig. 3B), phage A8 was observed to share high similarity to phage A10 (coverage: 97%; identity: 92%) but little or low similarity to other phages.

Further phylogenetic analysis was conducted for the phages isolated in this study to determine their taxonomic relationship. As shown in Fig. 3, phages isolated in this study clustered to 3 different phage families, namely *Autographiviridae*, *Tectiviridae* and *Myoviridae*. Within the *Autographiviridae* phage family, *E. coli* K12 phage A7 is taxonomically clustered to phage genus *Teseptimavirus*, whilst *P. putida* KT2440 phage A11 is taxonomically clustered to phage genus *Ghunavirus*. For *P. putida* KT2440 phages A8 and A10, no taxonomically known phage genus is found associated with these two phages. *P. putida* KT2440 phage A9 is the only phage taxonomically associated within the *Tectiviridae* phage family, under phage genus *Alphatectiviridae*. *E. coli* K12 phage A1 and A6 are taxonomically clustered to phage genus *Felixounavirus*, whilst *E. coli* K12 phage A2 is taxonomically clustered to phage genus *Mosigvirus*.

### Protein abundance related to *E. coli* K12 phage receptors

**Fig. 4:**
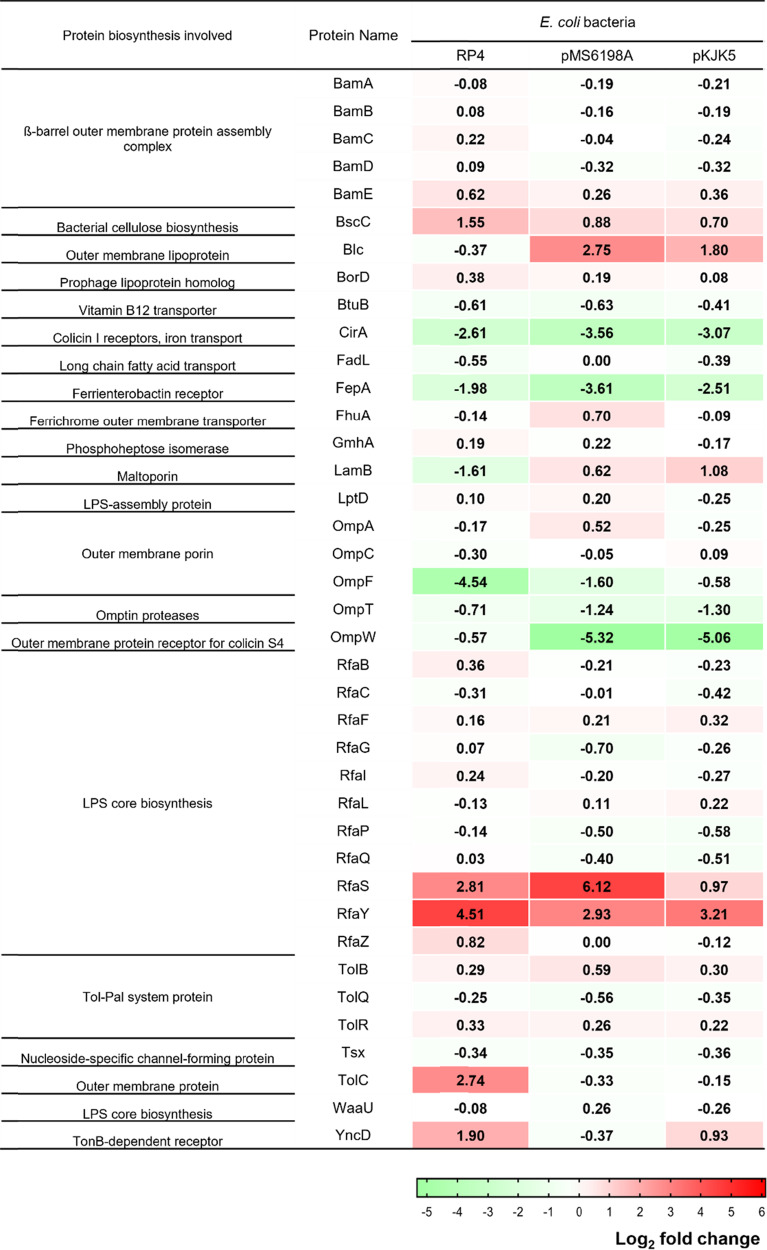
Log_2_ fold change of membrane proteins of *E. coli* K12 bacteria harbouring different plasmids in comparison to *E. coli* K12 wild type (no plasmid). RP4 represents *E. coli* K12 harbouring plasmid RP4; pMS6198A represents *E. coli* K12 harbouring plasmid pMS6198A; pKJK5 represents *E. coli* K12 bacteria harbouring plasmid pKJK5.

Total protein sequencing of *E. coli* K12 bacteria was conducted to evaluate the expression of bacterial protein under the influence of different plasmids compared to wild type strain (containing no plasmid). Specifically, bacterial membrane proteins that were previously identified as potential cell surface receptors for phage were selected for analysis [26, 27]. The fold change value reported for the membrane protein expression as shown in Fig. 4 is log_2_ transformed. From the total 39 phage-receptor associated bacterial membrane protein identified, the abundance of membrane protein RfaS and RfaY were found to have a high log_2_ fold change ranging from 0.97 to 6.12 fold across all 3 different *E. coli* K12 bacteria harbouring different plasmids. Apart from the high abundance of membrane proteins observed, membrane protein OmpF observed in *E. coli* K12 harbouring plasmids RP4 and OmpW observed in *E. coli* K12 bacteria harbouring plasmids pMS6198A and pKJK5 were also observed to express the lowest abundance fold change in comparison to wild type *E. coli* K12 bacteria.

### Identification of phage receptors for *E. coli* K12 phages

Phage resistant mutants were generated to screen for genomic variants before and after exposure to phage treatment. Table 3 summarizes the genomic variants identified in phage-resistant mutants post-exposure to different *E. coli* K12 phages. Phage-resistant mutants exposed to both phages A1 and A6 were found to have mutations in the gene *waaU*. Specifically, phage A6-resistant mutants were found to have multiple nucleotide insertion mutations whereas phage A1-resistant mutants were found to have single nucleotide deletion mutations. Phage A2-resistant mutants were found to have single nucleotide substitution mutations observed in the gene *ompC*. Meanwhile, phage A7-resistant mutants were found to have two nucleotide deletion mutations observed in the gene *hldE*. In this study, although the same genomic mutational change was observed in all 3 biological triplicate of phage-resistant mutant colonies generated, further studies using population-based sequencing are recommended which would allow a high-resolution analysis identifying mutational change in these phage-resistant mutants.

**Table 3:** Summary of genomic mutational changes observed in phage-resistant mutants.

Phage	Mutant strains derived from	Gene	Mutational change	Functional role of gene
vB_EcoM_LNA1	*E. coli* K12 MG1655 w/ plasmid RP4	*waaU*	Single nucleotide deletion	Lipopolysaccharide core biosynthesis
vB_EcoM_LNA2	*E. coli* K12 MG1655 wild type	*ompC*	Single nucleotide substitution	Outer membrane protein
vB_EcoM_LNA6	*E. coli* K12 MG1655 wild type	*waaU*	Multiple nucleotide insertion	Lipopolysaccharide (LPS) core biosynthesis
vB_EcoM_LNA7	*E. coli* K12 MG1655 w/ plasmid pKJK5	*hldE*	Two nucleotide deletion	Lipopolysaccharide core biosynthesis

### Expanded phage adsorption assay

Expanded phage adsorption assays were conducted following the analysis of bacterial membrane protein, which showed increased expression of LPS-related protein in plasmid harbouring *E. coli* K12 bacteria. Specifically, *E. coli* K12 phage A1 and phage A2 were selected to compare adsorption efficiency in the original bacteria host (bacteria host where phage were originally isolated from) as well as wild type *E. coli* K12 (containing no plasmid). The results are shown in Fig. S14. From the result, we’ve observed different phage adsorption efficiency for both phages A1 and A2, in terms of maximum phage adorption percentage and time required to achieve maximum adsorption. For phage A1, maximum phage adsorption (99.3 ± 0.3%) were observed in 20 min in the original bacteria host (*E.coli* K12 harbouring plasmid RP4) whilst it took 30 min to achieve maximum adsorption (88.4 ± 1.2%) in wild type *E.coli* K12 (Fig. S14A). Similar phenomena were also observed for phage A2 where maximum phage adsorption (97.9 ± 0.3%) were observed in 20 min in the original bacteria host (*E. coli* K12 harbouring plasmid pMS6198A plasmid), whilst it took 30 min to achieve maximum adsorption (95.8 ± 0.1%) in wild type *E. coli* K12 (Fig. S14B).

## Discussion

### Physiological and genomic traits of isolated phage

In this study, two different genera of bacteria (*E. coli* K12 and *P. putida* KT2440) were modified to harbour different plasmids and were used for subsequent phage isolation and characterisation. Despite the similarity observed in phage morphologies, subsequent phage physiological characterisation and genomic characterisation provided evidence that differentiated the individual phages isolated in this study. Interestingly, *P. putida* KT2440 phage A8 and A10 are taxonomically clustered in an unassigned phage genus clade within the phage family *Autographiviridae*, suggesting that both phages might be new phage species members under a new phage genus. Additionally, the phages isolated in this study were predicted to adopt strictly phage lytic lifestyles except for phage A8, which was predicted to adopt a temperate lifestyle. Although features of temperate phage such as integrase or transposon related genes were not found, phage A8 was found to encode a unique 543 bp putative hypothetical protein which was not found in phage A10, which shares high whole genome similarity. It is postulated that this putative hypothetical protein might contribute towards the *in silico* predicted temperate phage lifestyle. Unlike the lytic phages, the temperate phages which have been reported carrying integrase or transposon related genes can adopt both lytic and lysogenic lifestyles [39], with the latter associated with reported elevated bacteria pathogenicity, as well as the spread of antibiotic resistance through transduction [40, 41]. Further experiments, such as using knockout phage mutants, are needed to understand the functional role of this protein and its correlation with phage replication lifestyle.

### Bacterial host range spectrum and infectivity of isolated phages

Phage spot test and EOP assays were conducted to investigate the host range spectrum and infectivity of the isolated phages. From the results, both *E. coli* K12 phages, as well as *P. putida* KT2440 phages, were found to infect bacteria within the same genus except for *P. putida* KT2440 phage A9, which can infect both *E. coli* K12 bacteria and *P. putida* KT2440 bacteria harbouring the IncP-1 conjugative plasmid (including plasmid RP4 or pKJK5). The observed plasmid-dependent behaviour for *P. putida* KT2440 phage A9 suggests that this phage is a plasmid-dependent phage. Furthermore, *P. putida* KT2440 phage A9 was also found to taxonomically cluster with other plasmid-dependent phage species member, such as phage PRD1 [42] within the phage family *Tectiviridae*. Plasmid-dependent phage represents a unique phage type that recognizes plasmid-encoded sex apparatus (i.e. pilus) as the receptor for gain of entrance into the bacterial cell. Such phage type has also been previously reported as potential novel phage candidates that limit the spread of antibiotic resistance by interfering the conjugational transfer of antibiotic resistance plasmid within a bacterial population [43, 44].

In conjunction with spot test assays, further EOP assays revealed that *E. coli* K12 phages and *P. putida* KT2440 phages were found to exhibit varying EOP values in other permissive bacteria host when compared to its original bacteria host (bacteria host use for phage isolation). Interestingly, *E. coli* K12 phage A7 was unable to produce plaque formations in some of the permissive bacteria host indicated via spot test assays. This observed phenomenon is likely due to the potential effect of phage lysis from without, where high multiplicity of phage adsorption on a bacteria host leads to premature bacterial cell lysis with no phage production [23]. The varying phage infectivity observed for the isolated phages against the same bacterial strain represents an interesting phenomenon. Although insights into the effect of plasmids affecting phage virulence are unclear, previous studies have shown that a plasmid encoded toxin-antitoxin system can affect phage virulence, often by inhibiting phage replication subsequently leading to bacterial abortive infection [14, 15, 45]. The plasmids introduced in our study have been known to carry TA systems such as parDE Type II TA system (plasmid RP4) [46] as well as Tad-Ata TA system (plasmid pMS6198A) [21]. However, it is unlikely that these TA systems contribute to the difference in phage virulence as effective phage replication still occured as observed through plaque formations. Instead, it is hypothesized that the differences observed in phage virulence may be attributed by plasmid presence in the bacterial host, which affects phage population dynamics such as phage adsorption, latent period and the subsequent burst size that may have been affected when infecting against other permissive bacteria hosts.

### Plasmid-modulated bacterial membrane protein affecting phage infectivity

To further investigate the mechanisms behind the influence of plasmids that led to the differences in phage infectivity, the total bacteria protein profiling of *E. coli* K12 bacteria was performed to assess the abundance of protein expression influenced by plasmids. Specifically, membrane proteins of *E. coli* K12 bacteria that were known to be targets as phage receptors were selected for analysis. From the results, membrane proteins RfaS and RfaY were observed to be expressed in high abundance across all plasmid harbouring *E. coli* K12 bacteria. Both RfaS and RfaY protein are membrane proteins that have been reported to be involved in biogenesis of the lipopolysaccharide (LPS) core in *E. coli* K12 bacteria [47]. The high abundance of these LPS-related membrane proteins also correlated well with phage receptors identified for the *E. coli* K12 phages. Specifically, phage-resistant bacterial mutants generated post infection by the *E. coli* K12 phages were also found to have mutational change in genes involved in biosynthesis of LPS core (e.g. *waaU* and *hldE*). The mutational change observed in the bacterial cell surface receptors represents one of the common phage defence mechanisms that prevents the attachment of phage to bacteria, a critical step for initiation of phage infection [7, 8].

From the results, it is postulated that the modulation of the bacterial membrane protein by plasmid affects the adsorption of phage subsequently leading to varying phage infectivity. This hypothesis is further confirmed through expanded phage adsorption assays of *E. coli* K12 phage A1 and phage A2, which displayed reduced phage adsorption efficiency when infecting wild type *E. coli* K12 that expresses low abundance of LPS-core related protein. Although *E. coli* K12 phage A2 was identified using OmpC as potential phage receptors, the reduced phage adsorption efficiency observed suggests that phage A2 may have utilized LPS-core related protein for reversible adsorption and OmpC for its final irreversible attachment. A similar phage adsorption process has also been reported for other phages such as phage T5, which uses LPS O-antigen for the initial reversible adsorption followed by irreversible attachment to outer membrane FhuA [48] . Phage adsorption represents a critical step for initiation of phage infection. Dependent on the accessibility and location of the bacterial cell surface receptors, phage ability to effectively infect a bacteria host can also vary [27]. Apart from the observed difference in phage adsorption efficiency, further studies investigating phage infectivity parameter such as one-step growth curve are proposed to better understand the underlying effect of plasmid towards phage virulence.

### The effect of plasmid towards phage within the context of microbial ecology and future implications

**Fig. 5:**
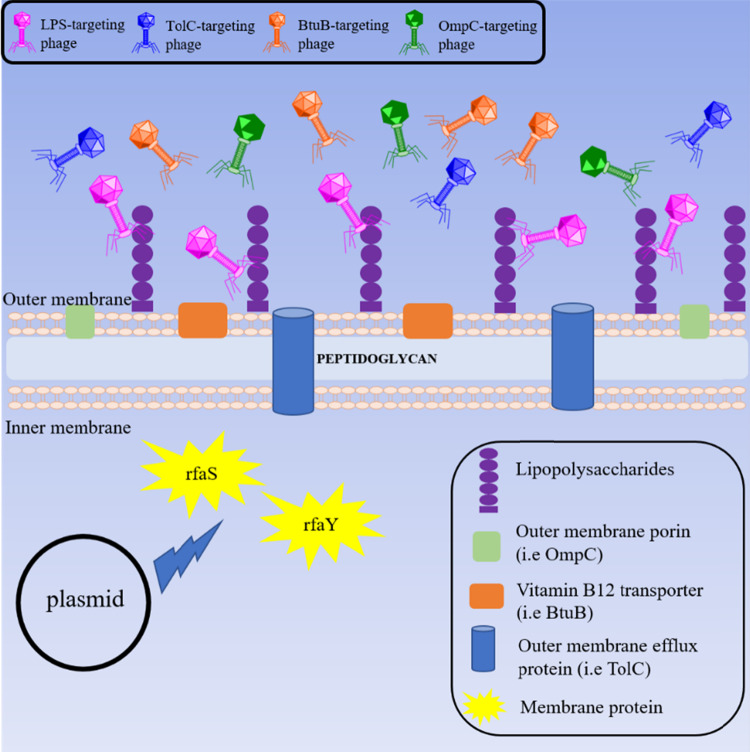
Schematic diagram illustrating the effect of plasmid that indirectly interferes with the expression of cell membrane protein RfaS and RfaY, related to biogenesis of LPS. Changes in the expression of these membrane proteins indirectly affect the expression of LPS cell surface receptors, thereby attracting phage that potentially utilize LPS as cell surface receptors.

The evolutionary arms race of phage and bacteria represents a major drive that shapes the ecology of both bacterial and phage populations in the environment [49]. Within the context of microbial ecology, the findings observed in our study highlight the unexplored effects of plasmids, which might act as a selective pressure to influence the diversity of phage population in a given niche. Specifically, our study shows that the presence of plasmid in bacteria influences the expression of the bacterial membrane proteins, which act as potential bacterial cell surface receptors recognised by phages, as illustrated in Fig. 5.

Additionally, the modulation of bacterial cell surface receptors by phage could also impact the adsorption efficiency of phage, which leads to varying phage infectivity outcome. Given the ubiquitous nature of plasmids, it is postulated that plasmids may have the potential to alter the physiological properties of bacteria with regards to the outer membrane structure, which could affect phage infectivity but also selects for phage that utilize plasmid-affected bacterial cell surface as receptors. It is also worthwhile to note that our study only isolated one phage type per bacteria strain tested, with limited plasmid types introduced. To strengthen the impact of the findings, further studies involving isolating multiple phages per bacteria strain with different plasmid types are required to determine the effect of plasmid presence towards phage isolation and infectivity.

## Conclusions

Although previous studies have demonstrated the role of plasmids to mediate phage resistance, the influence of plasmids towards other aspects of phage dynamics, such as isolation and subsequent infectivity, remains unknown. In this study, phages with different physiological and genomic traits were isolated using two different groups of bacteria containing different plasmids. Interestingly, these phages were also found to have varying infection efficiency against the same bacteria harbouring different plasmids. The effects of plasmids towards alteration of bacterial membrane proteins and the subsequent cell surface receptor identification for isolated phages also revealed the potential correlation of plasmids and the phage isolation process. Further in-depth studies are still needed to fully understand the influence of plasmids towards overall expression of bacteria membrane proteins, as well as their effects on phage isolation and replication.

## Supplementary information

Supporting Information

## Data Availability

The complete genomic sequence of the phage vB_EcoM_LNA1, vB_EcoM_LNA2, vB_EcoM_LNA6, vB_EcoP_LNA7, vB_PputP_LNA8, vB_PputP_LNA9, vB_PputP_LNA10 and vB_PputP_LNA11 were individually submitted in the NCBI database under the accession numbers MZ311863, MZ311865, OM630585, OM630586, OM630587, OM630588, OM630589 and OM630590. Meanwhile the genomic sequence data for phage resistant mutants generated in this study were submitted under accession numbers CP097884 (phage vB_EcoM_LNA1 resistant mutant), CP097883 (phage vB_EcoM_LNA2 resistant mutant), CP097882 (phage vB_EcoM_LNA6 resistant mutant) and CP097881 (phage vB_EcoP_LNA7 resistant mutant).
