# Supplementary material for: The presence of plasmids in bacterial hosts alters phage isolation and infectivity
Source: ISME Commun. 2022 Aug 19;2:75. doi: 10.1038/s43705-022-00158-9 (PMC9723711; doi:10.1038/s43705-022-00158-9)
Supplement: Supplementary file 1 — Supporting Information [file 43705_2022_158_MOESM1_ESM.docx]

**Supporting Information**

**The presence of plasmids in bacterial hosts alters phage isolation and infectivity**

Lyman Ngiam ^a^, Karen D. Weynberg* ^b^, Jianhua Guo* ^a^

^a^ Australian Centre for Water and Environmental Biotechnology, University of Queensland, Brisbane, Australia

^b^ Australian Centre for Ecogenomics, School of Chemistry and Molecular Biosciences, University of Queensland, Brisbane, Australia

*These authors have contributed equally to this work

Corresponding author: [jianhua.guo@uq.edu.au](mailto:jianhua.guo@uq.edu.au), Gehrmann Laboratories Building, The University of Queensland, Research Rd, St Lucia QLD 4067, +61 7 3346 3222.

**This file includes:**

Supplementary Text 1 to 2

Supplementary Tables 1 to 13

Supplementary Figures 1 to 16

**Text S1. Protein extractions and proteomic analysis**

Prior to total protein extraction, fresh overnight grown bacteria was prepared. Next, total protein of bacteria was extracted as described previously (Wang et al., 2019). Briefly, the total bacterial protein was extracted from pelleted cells using the B-PER method, followed by reduction, alkylation, digestion, and ziptip clean-up procedures. Following this extraction, 5 μg aliquot of the purified protein from each triplicate sample were applied for mass spectrometry analysis and for construction of protein libraries by information dependent analysis (IDA). Then, another 1 μg aliquot of the purified protein from each triplicate sample were used for SWATH-MS analysis. Samples were applied to a Triple-T of 5600 instrument (ABSciex, USA) equipped with a Nanospray III interface, using the settings as described previously (Grobbler et al., 2015).

IDA data were combined and searched using ProteinPilot software, with the combined

databases of *E. coli* K12 proteome (proteome ID: UP000000625; received from Uniprot on 23^rd^ of August 2021). Search setting for enzyme digestion was set to Trypsin and alkylation was set to iodoacetamide. Afterwards, the constructed IDA library and SWATH-MS data were loaded into PeakView v2.1 for further processing, with the peptide confidence threshold of 99%, number of peptides per protein of 5, and number of transitions per peptide of 3. A minimum of 2 peptides and 3 transitions was used for quantitative analysis.

**Text S2. Processing raw Illumina and Nanopore data for hybrid assembly using Unicycler**

Raw reads received were trimmed and filtered by using Trimmomatic (Bolger et al., 2014) to remove adaptor sequences and reads less than 36 bp when a sliding window of 4 bp and minimum Phred score of 30 was applied. Meanwhile, raw data generated from Nanopore sequencing in Fast5 format were basecalled and converted in Fastq format using Guppy neural network basecalling software (Wick et al., 2019).

**Table S1**. Details of plasmids used in this study

| **Plasmid** | **Plasmid type** | **Harbored antibiotic resistance gene** | **Reference** |
| --- | --- | --- | --- |
| RP4 | lncP-α; conjugative | Ampicillin, Kanamycin and Tetracycline | (Pansegrau et al., 1994) |
| pMS6198A | lncA/C2; conjugative | Multiple antibiotic resistance gene including bla_CMY_ and bla_NDM_ | (Hancock et al., 2017) |
| pKJK5 | lncP-1; conjugative | Tetracycline, trimethoprim and spectinomycin | (Bahl et al., 2007) |

**Table S2**. EOP of isolated phage against the different bacteria host. NA indicates non-permissive host based on previous spot test assay. N.D indicates no plaque formation were observed. Each value is presented as mean values ± standard deviation from three independent experiments.

| **Phage** | | **Bacteria host** | | | | | | | | | | | | | | |
| --- | --- | --- | --- | --- | --- | --- | --- | --- | --- | --- | --- | --- | --- | --- | --- | --- |
|  |  | ***E. coli* K12 MG1655** | | | | | | | ***P. putida* KT2440** | | | | | | | |
|  |  | **WT** | | **RP4 plasmid** | | **pMS6198A plasmid** | | **pKJK5 plasmid** | **WT** | | **RP4 plasmid** | | **pMS6198A plasmid** | | **pKJK5 plasmid** | |
| **A1** | 0.70 ± 0.10 | | 1.0 ± 0.0 | | 0.67 ± 0.07 | | 0.53 ± 0.05 | | | NA | | NA | | NA | | NA |
| **A2** | 0.50 ± 0.06 | | 0.84 ± 0.04 | | 1.0 ± 0.0 | | 0.73 ± 0.03 | | | NA | | NA | | NA | | NA |
| **A6** | 1.0 ± 0.0 | | 0.11 ± 0.02 | | 0.69 ± 0.07 | | 0.68 ± 0.06 | | | NA | | NA | | NA | | NA |
| **A7** | N.D | | N.D | | 0.35 ± 0.04 | | 1.0 ± 0.0 | | | NA | | NA | | NA | | NA |
| **A8** | NA | | NA | | NA | | NA | | | 1.0 ± 0.0 | | 0.07 ± 0.03 | | 0.53 ± 0.03 | | 1.34 ± 0.19 |
| **A9** | NA | | 0.08 ± 0.01 | | NA | | 0.25 ± 0.09 | | | NA | | 1.0 ± 0.0 | | NA | | 0.49 ± 0.05 |
| **A10** | NA | | NA | | NA | | NA | | | 1.57 ± 0.04 | | 0.21 ± 0.07 | | 1.0 ± 0.0 | | 2.23 ± 0.11 |
| **A11** | NA | | NA | | NA | | NA | | | 0.52 ± 0.16 | | 0.56 ± 0.18 | | 0.47 ± 0.13 | | 1.0 ± 0.0 |

**Table S3.** PHACTS phage lifestyle prediction for phages and the associated probability

| **Phage** | **Predicted lifestyle** | **Probability** | **Standard Deviation** |
| --- | --- | --- | --- |
| A1 | Confidently lytic | 0.562 | 0.048 |
| A2 | Confidently lytic | 0.776 | 0.033 |
| A6 | Confidently lytic | 0.539 | 0.049 |
| A7 | Confidently lytic | 0.744 | 0.031 |
| A8 | Non-confidently temperate | 0.536 | 0.053 |
| A9 | Confidently lytic | 0.527 | 0.037 |
| A10 | Confidently lytic | 0.525 | 0.048 |
| A11 | Confidently lytic | 0.623 | 0.053 |

Table S4. List of genome features of phage A1

| Name | Type | Start | Stop | Length | Direction |
| --- | --- | --- | --- | --- | --- |
| hypothetical protein | CDS | 811 | 1359 | 549 | reverse |
| hypothetical protein | CDS | 1373 | 1837 | 465 | reverse |
| hypothetical protein | CDS | 1890 | 2114 | 225 | reverse |
| hypothetical protein | CDS | 2191 | 2694 | 504 | reverse |
| hypothetical protein | CDS | 2691 | 2909 | 219 | reverse |
| hypothetical protein | CDS | 2993 | 3574 | 582 | reverse |
| hypothetical protein | CDS | 3574 | 3918 | 345 | reverse |
| hypothetical protein | CDS | 3911 | 4204 | 294 | reverse |
| D-alanyl-D-alanine carboxypeptidase | CDS | 4204 | 4599 | 396 | reverse |
| hypothetical protein | CDS | 4592 | 4990 | 399 | reverse |
| Lysozyme | CDS | 5043 | 5507 | 465 | reverse |
| putative tail protein | CDS | 5507 | 6892 | 1386 | reverse |
| hypothetical protein | CDS | 6889 | 7260 | 372 | reverse |
| hypothetical protein | CDS | 7309 | 7899 | 591 | reverse |
| hypothetical protein | CDS | 8519 | 8890 | 372 | forward |
| hypothetical protein | CDS | 8972 | 10387 | 1416 | forward |
| tRNA-Pro | tRNA | 10554 | 10630 | 77 | forward |
| tRNA-Glu | tRNA | 10638 | 10715 | 78 | forward |
| tRNA-Asn | tRNA | 10968 | 11044 | 77 | forward |
| tRNA-Tyr | tRNA | 11054 | 11141 | 88 | forward |
| tRNA-Lys | tRNA | 11570 | 11645 | 76 | forward |
| tRNA-Met | tRNA | 11650 | 11726 | 77 | forward |
| tRNA-lle | tRNA | 11729 | 11804 | 76 | forward |
| hypothetical protein | CDS | 12369 | 12773 | 405 | forward |
| tRNA-Ser | tRNA | 12787 | 12874 | 88 | forward |
| tRNA-Leu | tRNA | 13131 | 13208 | 78 | forward |
| tRNA-Lys | tRNA | 13216 | 13291 | 76 | forward |
| tRNA-Ala | tRNA | 13298 | 13373 | 76 | forward |
| tRNA-Gly | tRNA | 13380 | 13454 | 75 | forward |
| tRNA-Thr | tRNA | 13461 | 13537 | 77 | forward |
| tRNA-Val | tRNA | 13633 | 13707 | 75 | forward |
| tRNA-Leu | tRNA | 13709 | 13786 | 78 | forward |
| hypothetical protein | CDS | 13806 | 14363 | 558 | forward |
| tRNA-Gln | tRNA | 14373 | 14447 | 75 | forward |
| tRNA-Leu | tRNA | 14450 | 14528 | 79 | forward |
| tRNA-Gln | tRNA | 14534 | 14609 | 76 | forward |
| tRNA-His | tRNA | 14641 | 14716 | 76 | forward |
| tRNA-Phe | tRNA | 14731 | 14798 | 68 | forward |
| hypothetical protein | CDS | 14815 | 15549 | 735 | forward |
| tRNA-Cys | tRNA | 15649 | 15724 | 76 | forward |
| hypothetical protein | CDS | 15947 | 16147 | 201 | forward |
| Terminase large subunit | CDS | 16169 | 17770 | 1602 | forward |
| hypothetical protein | CDS | 17787 | 19253 | 1467 | forward |
| hypothetical protein | CDS | 19253 | 19753 | 501 | forward |
| hypothetical protein | CDS | 19753 | 20085 | 333 | forward |
| Head maturation protease | CDS | 20097 | 21443 | 1347 | forward |
| Phage structural protein | CDS | 21455 | 21832 | 378 | forward |
| Major capsid protein | CDS | 21866 | 22972 | 1107 | forward |
| hypothetical protein | CDS | 22994 | 23443 | 450 | forward |
| hypothetical protein | CDS | 23443 | 23925 | 483 | forward |
| hypothetical protein | CDS | 23922 | 24323 | 402 | forward |
| hypothetical protein | CDS | 24298 | 24897 | 600 | forward |
| hypothetical protein | CDS | 24898 | 26250 | 1353 | forward |
| hypothetical protein | CDS | 26266 | 26712 | 447 | forward |
| Tape measure chaperone | CDS | 26786 | 27184 | 399 | forward |
| hypothetical protein | CDS | 27187 | 27426 | 240 | forward |
| Putative tail length tape measure protein | CDS | 27426 | 29669 | 2244 | forward |
| hypothetical protein | CDS | 29669 | 30466 | 798 | forward |
| hypothetical protein | CDS | 30466 | 30807 | 342 | forward |
| hypothetical protein | CDS | 30807 | 31784 | 978 | forward |
| Baseplate assembly protein | CDS | 31784 | 32407 | 624 | forward |
| hypothetical protein | CDS | 32407 | 32826 | 420 | forward |
| Baseplate assembly protein | CDS | 32826 | 34295 | 1470 | forward |
| Putative baseplate assembly protein | CDS | 34298 | 35155 | 858 | forward |
| hypothetical protein | CDS | 35155 | 35457 | 303 | forward |
| Tail fibre protein | CDS | 35460 | 36608 | 1149 | forward |
| Tail fiber proteins | CDS | 36655 | 39006 | 2352 | forward |
| hypothetical protein | CDS | 39086 | 39280 | 195 | forward |
| Holin | CDS | 39281 | 39652 | 372 | forward |
| Thymidylate synthase | CDS | 39689 | 40588 | 900 | forward |
| Dihydrofolate reductase | CDS | 40590 | 41135 | 546 | forward |
| hypothetical protein | CDS | 41393 | 41908 | 516 | reverse |
| hypothetical protein | CDS | 41922 | 42281 | 360 | reverse |
| Phage repressor | CDS | 42283 | 42582 | 300 | forward |
| hypothetical protein | CDS | 42575 | 42790 | 216 | reverse |
| hypothetical protein | CDS | 42792 | 43175 | 384 | reverse |
| DNA ligase | CDS | 43463 | 44563 | 1101 | forward |
| hypothetical protein | CDS | 44560 | 44766 | 207 | reverse |
| hypothetical protein | CDS | 44777 | 45034 | 258 | reverse |
| hypothetical protein | CDS | 45024 | 45170 | 147 | reverse |
| hypothetical protein | CDS | 45167 | 45385 | 219 | reverse |
| hypothetical protein | CDS | 45382 | 45591 | 210 | reverse |
| HNH endonuclease | CDS | 45643 | 46179 | 537 | reverse |
| DNA polymerase | CDS | 46166 | 46720 | 555 | reverse |
| hypothetical protein | CDS | 46950 | 49133 | 2184 | reverse |
| hypothetical protein | CDS | 49392 | 49814 | 423 | forward |
| Minor tail protein | CDS | 49816 | 50628 | 813 | forward |
| Putative deoxynucleotide monophosphate kinase | CDS | 50690 | 51433 | 744 | forward |
| hypothetical protein | CDS | 51448 | 51642 | 195 | forward |
| DNA primase/helicase | CDS | 51635 | 53620 | 1986 | forward |
| hypothetical protein | CDS | 53873 | 54022 | 150 | forward |
| hypothetical protein | CDS | 54095 | 54952 | 858 | forward |
| Exodeoxyribonuclease | CDS | 55015 | 56055 | 1041 | forward |
| hypothetical protein | CDS | 56045 | 56542 | 498 | forward |
| hypothetical protein | CDS | 56564 | 56812 | 249 | forward |
| hypothetical protein | CDS | 56788 | 57543 | 756 | forward |
| hypothetical protein | CDS | 57524 | 57847 | 324 | forward |
| hypothetical protein | CDS | 57840 | 58175 | 336 | forward |
| Ribonucleoside triphosphate reductase large subunit | CDS | 58222 | 60456 | 2235 | forward |
| hypothetical protein | CDS | 60599 | 60769 | 171 | forward |
| Ribonucleoside triphosphate reductase small subunit | CDS | 60766 | 61839 | 1074 | forward |
| Putative glutaredoxin | CDS | 61839 | 62081 | 243 | forward |
| Putative membrane protein | CDS | 62074 | 62280 | 207 | forward |
| Putative anaerobic ribonucleoside-triphosphate reductase | CDS | 62329 | 63528 | 1200 | forward |
| Homing endonuclease | CDS | 63641 | 64105 | 465 | forward |
| Putative anaerobic nucleoside diphosphate reductase | CDS | 64361 | 65215 | 855 | forward |
| hypothetical protein | CDS | 65814 | 66209 | 396 | forward |
| hypothetical protein | CDS | 66206 | 66505 | 300 | forward |
| Putative anaerobic nucleoside-triphosphate reductase small subunit | CDS | 66515 | 67000 | 486 | forward |
| hypothetical protein | CDS | 66963 | 67340 | 378 | forward |
| hypothetical protein | CDS | 67307 | 67564 | 258 | forward |
| hypothetical protein | CDS | 67567 | 67887 | 321 | forward |
| hypothetical protein | CDS | 67939 | 68454 | 516 | forward |
| hypothetical protein | CDS | 68447 | 68725 | 279 | forward |
| Ribose-phosphate pyrophosphokinase | CDS | 68737 | 69618 | 882 | forward |
| Nicotinamide phosphoribosyltransferase | CDS | 69664 | 71445 | 1782 | forward |
| hypothetical protein | CDS | 71499 | 71834 | 336 | forward |
| hypothetical protein | CDS | 71816 | 71992 | 177 | forward |
| Phage rIIA lysis inhibitor | CDS | 72021 | 74387 | 2367 | forward |
| rIIB protein | CDS | 74467 | 75576 | 1110 | forward |
| hypothetical protein | CDS | 75677 | 76225 | 549 | forward |
| hypothetical protein | CDS | 76203 | 76898 | 696 | forward |
| hypothetical protein | CDS | 76909 | 77373 | 465 | forward |
| hypothetical protein | CDS | 77424 | 78308 | 885 | forward |
| hypothetical protein | CDS | 78320 | 79132 | 813 | forward |
| hypothetical protein | CDS | 79179 | 79526 | 348 | forward |
| hypothetical protein | CDS | 79492 | 79689 | 198 | forward |
| hypothetical protein | CDS | 79755 | 79955 | 201 | forward |
| hypothetical protein | CDS | 79958 | 80281 | 324 | forward |
| hypothetical protein | CDS | 80259 | 80486 | 228 | forward |
| hypothetical protein | CDS | 80499 | 80699 | 201 | forward |
| Phosphatase | CDS | 80700 | 81485 | 786 | forward |
| hypothetical protein | CDS | 82007 | 82195 | 189 | forward |
| hypothetical protein | CDS | 82493 | 82873 | 381 | forward |
| hypothetical protein | CDS | 82962 | 83258 | 297 | forward |
| hypothetical protein | CDS | 83252 | 83584 | 333 | forward |
| hypothetical protein | CDS | 83675 | 83947 | 273 | forward |
| hypothetical protein | CDS | 84034 | 84438 | 405 | forward |
| hypothetical protein | CDS | 84537 | 84740 | 204 | forward |
| hypothetical protein | CDS | 84826 | 85368 | 543 | forward |
| hypothetical protein | CDS | 85435 | 85668 | 234 | forward |
| hypothetical protein | CDS | 85760 | 86101 | 342 | forward |
| hypothetical protein | CDS | 86324 | 86854 | 531 | forward |
| hypothetical protein | CDS | 86918 | 87181 | 264 | forward |
| hypothetical protein | CDS | 87247 | 87495 | 249 | forward |

# Table S5. List of genome features of phage A2

| Name | Type | Start | Stop | Length | Direction |
| --- | --- | --- | --- | --- | --- |
| hypothetical protein | CDS | 122 | 379 | 258 | forward |
| hypothetical protein | CDS | 366 | 605 | 240 | forward |
| Endonuclease | CDS | 602 | 2290 | 1689 | forward |
| hypothetical protein | CDS | 2345 | 2533 | 189 | forward |
| RNA polymerase binding protein | CDS | 2546 | 2962 | 417 | forward |
| Putative sliding clamp DNA polymerase accessory protein | CDS | 3005 | 3691 | 687 | forward |
| Putative clamp loader small subunit | CDS | 3767 | 4729 | 963 | forward |
| Clamp loader small subunit | CDS | 4731 | 5294 | 564 | forward |
| Translation repressor protein | CDS | 5297 | 5665 | 369 | forward |
| DNA polymerase | CDS | 5747 | 8458 | 2712 | forward |
| Arabinose 5-phosphate isomerase | CDS | 8499 | 9134 | 636 | forward |
| hypothetical protein | CDS | 9131 | 9274 | 144 | forward |
| hypothetical protein | CDS | 9316 | 11001 | 1686 | forward |
| hypothetical protein | CDS | 11001 | 11387 | 387 | forward |
| Putative peptidase | CDS | 11446 | 12606 | 1161 | forward |
| hypothetical protein | CDS | 12603 | 12752 | 150 | forward |
| hypothetical protein | CDS | 12885 | 13601 | 717 | forward |
| hypothetical protein | CDS | 13601 | 14500 | 900 | forward |
| hypothetical protein | CDS | 14502 | 15050 | 549 | forward |
| RecA-like recombination protein | CDS | 15150 | 16322 | 1173 | forward |
| Capsid and scaffold protein | CDS | 16315 | 16656 | 342 | forward |
| DNA helicase | CDS | 16666 | 18108 | 1443 | forward |
| hypothetical protein | CDS | 18197 | 18571 | 375 | forward |
| hypothetical protein | CDS | 18627 | 18944 | 318 | forward |
| hypothetical protein | CDS | 18941 | 19129 | 189 | forward |
| Putative membrane protein | CDS | 19201 | 19569 | 369 | forward |
| Immunity to superinfection membrane protein | CDS | 19631 | 19879 | 249 | forward |
| Spackle periplasmic protein | CDS | 19943 | 20236 | 294 | forward |
| hypothetical protein | CDS | 20238 | 20888 | 651 | forward |
| hypothetical protein | CDS | 20890 | 21087 | 198 | forward |
| hypothetical protein | CDS | 21107 | 21574 | 468 | forward |
| DNA primase subunit | CDS | 21614 | 22636 | 1023 | forward |
| hypothetical protein | CDS | 22633 | 22830 | 198 | reverse |
| dCTP pyrophosphatase | CDS | 22921 | 23442 | 522 | forward |
| Capsid and scaffold protein | CDS | 23489 | 23725 | 237 | forward |
| hypothetical protein | CDS | 23830 | 24057 | 228 | forward |
| hypothetical protein | CDS | 24023 | 24271 | 249 | forward |
| hypothetical protein | CDS | 24268 | 24447 | 180 | forward |
| hypothetical protein | CDS | 24447 | 24911 | 465 | forward |
| hypothetical protein | CDS | 24928 | 25092 | 165 | forward |
| hypothetical protein | CDS | 25089 | 25253 | 165 | forward |
| ADP-ribosylase | CDS | 25309 | 25890 | 582 | forward |
| ADP-ribosyltransferase | CDS | 25948 | 26556 | 609 | forward |
| anti-sigma factor | CDS | 26709 | 27455 | 747 | forward |
| hypothetical protein | CDS | 27458 | 27769 | 312 | forward |
| DNA helicase | CDS | 27766 | 29079 | 1314 | forward |
| Exonuclease A | CDS | 29089 | 29766 | 678 | forward |
| hypothetical protein | CDS | 29833 | 30327 | 495 | forward |
| Modifier of transcription | CDS | 30390 | 30887 | 498 | forward |
| MotB-like protein | CDS | 30897 | 31316 | 420 | forward |
| hypothetical protein | CDS | 31376 | 31900 | 525 | forward |
| hypothetical protein | CDS | 31958 | 32185 | 228 | forward |
| mRNA metabolism modulator | CDS | 32185 | 32598 | 414 | forward |
| Zinc ribbon domain protein | CDS | 32598 | 32777 | 180 | forward |
| hypothetical protein | CDS | 32780 | 33223 | 444 | forward |
| DNA topoisomerase II large subunit | CDS | 33287 | 35104 | 1818 | forward |
| hypothetical protein | CDS | 35147 | 36247 | 1101 | forward |
| hypothetical protein | CDS | 36355 | 36540 | 186 | forward |
| rIIA lysis inhibitor | CDS | 36553 | 38766 | 2214 | forward |
| rIIB lysis inhibitor | CDS | 38776 | 39723 | 948 | forward |
| hypothetical protein | CDS | 39765 | 40052 | 288 | forward |
| Endonuclease IV | CDS | 40069 | 40545 | 477 | forward |
| hypothetical protein | CDS | 40614 | 40877 | 264 | forward |
| hypothetical protein | CDS | 41128 | 41328 | 201 | forward |
| Nucleoid disruption protein | CDS | 41405 | 41851 | 447 | forward |
| hypothetical protein | CDS | 41904 | 42050 | 147 | forward |
| DNA topoisomerase II medium subunit | CDS | 42195 | 43520 | 1326 | forward |
| hypothetical protein | CDS | 43706 | 43921 | 216 | forward |
| activator of middle transcription | CDS | 44026 | 44658 | 633 | forward |
| hypothetical protein | CDS | 44669 | 44998 | 330 | forward |
| hypothetical protein | CDS | 44995 | 45456 | 462 | forward |
| hypothetical protein | CDS | 45456 | 45737 | 282 | forward |
| hypothetical protein | CDS | 45724 | 45939 | 216 | forward |
| hypothetical protein | CDS | 45914 | 46033 | 120 | forward |
| hypothetical protein | CDS | 46023 | 46322 | 300 | forward |
| hypothetical protein | CDS | 46312 | 46473 | 162 | forward |
| hypothetical protein | CDS | 46520 | 46792 | 273 | forward |
| Putative holin | CDS | 46793 | 47452 | 660 | reverse |
| Tail fibers protein | CDS | 47462 | 48013 | 552 | reverse |
| Long-tail fiber protein | CDS | 48044 | 51127 | 3084 | reverse |
| Putative tail fiber protein | CDS | 51136 | 51801 | 666 | reverse |
| Tail connector protein | CDS | 51864 | 52991 | 1128 | reverse |
| Long tail fiber proximal subunit | CDS | 53000 | 56878 | 3879 | reverse |
| Ribonuclease H | CDS | 56982 | 57899 | 918 | forward |
| dsDNA binding protein | CDS | 57907 | 58176 | 270 | forward |
| Late promoter transcription accessory protein | CDS | 58154 | 58492 | 339 | forward |
| DNA helicase loader | CDS | 58489 | 59142 | 654 | forward |
| single-stranded DNA binding protein | CDS | 59269 | 60171 | 903 | forward |
| FRD2 protein | CDS | 60285 | 60677 | 393 | forward |
| hypothetical protein | CDS | 60740 | 60988 | 249 | forward |
| hypothetical protein | CDS | 60991 | 61239 | 249 | forward |
| Dihydrofolate reductase | CDS | 61232 | 61819 | 588 | forward |
| Putative thymidylate synthase | CDS | 61816 | 62676 | 861 | forward |
| hypothetical protein | CDS | 62678 | 62932 | 255 | forward |
| Ribonucleoside-diphosphate reductase alpha | CDS | 63020 | 65275 | 2256 | forward |
| ribonucleotide reductase of class Ia | CDS | 65329 | 66507 | 1179 | forward |
| Endonuclease II | CDS | 66534 | 66944 | 411 | forward |
| RNA ligase A | CDS | 67001 | 68125 | 1125 | forward |
| Inhibitor of host transcription | CDS | 68125 | 68688 | 564 | forward |
| hypothetical protein | CDS | 68676 | 69032 | 357 | forward |
| Outer membrane lipoprotein | CDS | 69029 | 69319 | 291 | forward |
| hypothetical protein | CDS | 69316 | 69534 | 219 | forward |
| hypothetical protein | CDS | 69592 | 69891 | 300 | forward |
| hypothetical protein | CDS | 69891 | 70082 | 192 | forward |
| Polynucleotide kinase | CDS | 70079 | 70978 | 900 | forward |
| hypothetical protein | CDS | 70978 | 71169 | 192 | forward |
| hypothetical protein | CDS | 71159 | 71374 | 216 | forward |
| hypothetical protein | CDS | 71382 | 71657 | 276 | forward |
| hypothetical protein | CDS | 71718 | 71954 | 237 | forward |
| hypothetical protein | CDS | 71941 | 72066 | 126 | forward |
| 3-deoxy-7-phosphoheptulonate synthase | CDS | 72075 | 73067 | 993 | forward |
| deoxycytidylate deaminase | CDS | 73067 | 73648 | 582 | forward |
| hypothetical protein | CDS | 73650 | 73946 | 297 | forward |
| Head assembly chaperone protein | CDS | 74004 | 74336 | 333 | forward |
| Lysis inhibition protein | CDS | 74461 | 74709 | 249 | forward |
| hypothetical protein | CDS | 74978 | 75157 | 180 | forward |
| hypothetical protein | CDS | 75290 | 75658 | 369 | forward |
| hypothetical protein | CDS | 75733 | 76098 | 366 | forward |
| hypothetical protein | CDS | 76134 | 76748 | 615 | forward |
| hypothetical protein | CDS | 76803 | 77000 | 198 | forward |
| hypothetical protein | CDS | 76990 | 77208 | 219 | forward |
| hypothetical protein | CDS | 77201 | 77659 | 459 | forward |
| hypothetical protein | CDS | 77656 | 78471 | 816 | forward |
| hypothetical protein | CDS | 78481 | 78750 | 270 | forward |
| DNA ligase | CDS | 78747 | 80240 | 1494 | forward |
| hypothetical protein | CDS | 80240 | 80428 | 189 | forward |
| ADP-ribosyltransferase | CDS | 80484 | 82571 | 2088 | forward |
| hypothetical protein | CDS | 82630 | 82923 | 294 | forward |
| Baseplate tail tube initiator | CDS | 82956 | 83918 | 963 | reverse |
| Baseplate subunit | CDS | 83918 | 85027 | 1110 | reverse |
| Baseplate hub | CDS | 85036 | 86808 | 1773 | reverse |
| Baseplate hub distal subunit | CDS | 86805 | 87275 | 471 | reverse |
| Baseplate hub subunit | CDS | 87286 | 88458 | 1173 | reverse |
| Baseplate hub assembly protein | CDS | 88455 | 89207 | 753 | reverse |
| Baseplate hub subunit | CDS | 89255 | 89881 | 627 | forward |
| Baseplate wedge subunit | CDS | 89881 | 90279 | 399 | forward |
| Recombination, repair and ssDNA binding protein | CDS | 90303 | 90773 | 471 | forward |
| hypothetical protein | CDS | 90773 | 90997 | 225 | forward |
| hypothetical protein | CDS | 91030 | 91197 | 168 | forward |
| hypothetical protein | CDS | 91257 | 91490 | 234 | reverse |
| Helicase | CDS | 91516 | 93030 | 1515 | reverse |
| Inhibitor of prohead protease | CDS | 93081 | 93749 | 669 | forward |
| Head outer capsid protein | CDS | 93759 | 95174 | 1416 | forward |
| hypothetical protein | CDS | 95275 | 95469 | 195 | forward |
| hypothetical protein | CDS | 95466 | 95717 | 252 | forward |
| RNA ligase | CDS | 95837 | 96835 | 999 | forward |
| Capsid vertex protein | CDS | 96868 | 98151 | 1284 | reverse |
| hypothetical protein | CDS | 98252 | 98521 | 270 | forward |
| Major capsid protein | CDS | 98574 | 100142 | 1569 | reverse |
| Prohead core protein | CDS | 100160 | 100972 | 813 | reverse |
| Prohead core scaffolding protein and protease | CDS | 101006 | 101647 | 642 | reverse |
| Prohead core protein | CDS | 101647 | 102072 | 426 | reverse |
| Prohead core protein | CDS | 102072 | 102311 | 240 | reverse |
| Portal vertex protein | CDS | 102311 | 103882 | 1572 | reverse |
| Tail tube protein | CDS | 103967 | 104458 | 492 | reverse |
| Tail sheath protein | CDS | 104571 | 106553 | 1983 | reverse |
| Terminase large subunit | CDS | 106584 | 108419 | 1836 | reverse |
| Small terminase protein | CDS | 108403 | 108897 | 495 | reverse |
| Tail sheath stabilizer and completion protein | CDS | 108907 | 109731 | 825 | reverse |
| Neck protein | CDS | 109781 | 110545 | 765 | reverse |
| Head completion, neck hetero-dimeric protein | CDS | 110547 | 111473 | 927 | reverse |
| Putative fibritin | CDS | 111506 | 112954 | 1449 | reverse |
| Short tail fiber protein | CDS | 112954 | 114537 | 1584 | reverse |
| Baseplate wedge subunit and tail pin | CDS | 114534 | 115193 | 660 | reverse |
| Baseplate wedge subunit and tail pin | CDS | 115193 | 116998 | 1806 | reverse |
| Baseplate wedge tail fiber connector | CDS | 116998 | 117870 | 873 | reverse |
| baseplate wedge subunit | CDS | 117943 | 118947 | 1005 | reverse |
| baseplate wedge subunit | CDS | 118940 | 121993 | 3054 | reverse |
| baseplate wedge subunit | CDS | 122035 | 124008 | 1974 | reverse |
| hypothetical protein | CDS | 124017 | 124310 | 294 | reverse |
| hypothetical protein | CDS | 124313 | 124786 | 474 | reverse |
| baseplate hub subunit and tail lysozyme | CDS | 124832 | 126511 | 1680 | reverse |
| baseplate wedge subunit | CDS | 126565 | 127140 | 576 | reverse |
| Head completion protein | CDS | 127202 | 127651 | 450 | forward |
| DNA end protection during packaging | CDS | 127654 | 128475 | 822 | forward |
| tail completion and sheath stabilizer protein | CDS | 128578 | 129162 | 585 | forward |
| deoxynucleotide monophosphate kinase | CDS | 129216 | 129950 | 735 | forward |
| tail fiber assembly helper protein | CDS | 129955 | 130185 | 231 | forward |
| hypothetical protein | CDS | 130185 | 130640 | 456 | forward |
| internal head nuclease inhibitor protein | CDS | 130718 | 131005 | 288 | forward |
| hypothetical protein | CDS | 131076 | 131261 | 186 | forward |
| hypothetical protein | CDS | 131263 | 131625 | 363 | forward |
| hypothetical protein | CDS | 131622 | 131912 | 291 | forward |
| hypothetical protein | CDS | 131917 | 132432 | 516 | forward |
| tRNA-IIe2 | tRNA | 132512 | 132586 | 75 | forward |
| tRNA-Arg | tRNA | 132590 | 132666 | 77 | forward |
| hypothetical protein | CDS | 132685 | 133029 | 345 | forward |
| hypothetical protein | CDS | 133413 | 134039 | 627 | forward |
| hypothetical protein | CDS | 134149 | 134454 | 306 | forward |
| hypothetical protein | CDS | 134522 | 134686 | 165 | forward |
| hypothetical protein | CDS | 134733 | 134960 | 228 | forward |
| hypothetical protein | CDS | 135031 | 135624 | 594 | forward |
| hypothetical protein | CDS | 135674 | 136270 | 597 | forward |
| hypothetical protein | CDS | 136239 | 136634 | 396 | forward |
| hypothetical protein | CDS | 136613 | 136972 | 360 | forward |
| hypothetical protein | CDS | 136969 | 137274 | 306 | forward |
| hypothetical protein | CDS | 137284 | 137556 | 273 | forward |
| hypothetical protein | CDS | 137566 | 137763 | 198 | forward |
| hypothetical protein | CDS | 137826 | 138065 | 240 | forward |
| hypothetical protein | CDS | 138097 | 139050 | 954 | forward |
| hypothetical protein | CDS | 139119 | 139424 | 306 | forward |
| hypothetical protein | CDS | 139426 | 140112 | 687 | forward |
| hypothetical protein | CDS | 140112 | 140603 | 492 | forward |
| hypothetical protein | CDS | 140600 | 140836 | 237 | forward |
| Nudix hydrolase | CDS | 140826 | 141284 | 459 | forward |
| Lysozyme | CDS | 141319 | 141807 | 489 | forward |
| hypothetical protein | CDS | 141804 | 142085 | 282 | forward |
| Endonuclease V | CDS | 142144 | 142557 | 414 | forward |
| hypothetical protein | CDS | 142570 | 142824 | 255 | forward |
| hypothetical protein | CDS | 142889 | 143203 | 315 | forward |
| hypothetical protein | CDS | 143229 | 143558 | 330 | forward |
| hypothetical protein | CDS | 143732 | 144271 | 540 | forward |
| hypothetical protein | CDS | 144268 | 144576 | 309 | forward |
| hypothetical protein | CDS | 144583 | 144945 | 363 | forward |
| hypothetical protein | CDS | 144945 | 145169 | 225 | forward |
| hypothetical protein | CDS | 145159 | 145425 | 267 | forward |
| hypothetical protein | CDS | 145425 | 145724 | 300 | forward |
| RNA endonuclease | CDS | 145781 | 146239 | 459 | forward |
| hypothetical protein | CDS | 146248 | 146790 | 543 | forward |
| Valyl-tRNA synthetase modifier | CDS | 146796 | 147134 | 339 | forward |
| Macro domain protein | CDS | 147214 | 147594 | 381 | forward |
| hypothetical protein | CDS | 147591 | 147803 | 213 | forward |
| hypothetical protein | CDS | 147800 | 148006 | 207 | forward |
| hypothetical protein | CDS | 148003 | 148185 | 183 | forward |
| Thymidine kinase | CDS | 148195 | 148776 | 582 | forward |
| hypothetical protein | CDS | 148804 | 149016 | 213 | forward |
| Lysis inhibition regulator | CDS | 149059 | 149331 | 273 | forward |
| hypothetical protein | CDS | 149433 | 149612 | 180 | forward |
| hypothetical protein | CDS | 149620 | 149829 | 210 | forward |
| hypothetical protein | CDS | 149994 | 150173 | 180 | forward |
| hypothetical protein | CDS | 150175 | 150705 | 531 | forward |
| hypothetical protein | CDS | 150715 | 151188 | 474 | forward |
| Thioredoxin | CDS | 151188 | 152174 | 987 | forward |
| hypothetical protein | CDS | 152206 | 152487 | 282 | forward |
| hypothetical protein | CDS | 152596 | 152814 | 219 | forward |
| hypothetical protein | CDS | 152884 | 153864 | 981 | forward |
| hypothetical protein | CDS | 154003 | 154290 | 288 | forward |
| hypothetical protein | CDS | 154349 | 154876 | 528 | forward |
| hypothetical protein | CDS | 154939 | 155934 | 996 | forward |
| hypothetical protein | CDS | 155990 | 156949 | 960 | forward |
| hypothetical protein | CDS | 157012 | 157962 | 951 | forward |
| hypothetical protein | CDS | 157962 | 158267 | 306 | forward |
| hypothetical protein | CDS | 158267 | 158680 | 414 | forward |
| Thioredoxin | CDS | 158673 | 158936 | 264 | forward |
| hypothetical protein | CDS | 158933 | 159148 | 216 | forward |
| hypothetical protein | CDS | 159151 | 159321 | 171 | forward |
| hypothetical protein | CDS | 159293 | 159454 | 162 | forward |
| Putative peptidase inhibitor | CDS | 159441 | 159854 | 414 | forward |
| hypothetical protein | CDS | 159864 | 160043 | 180 | forward |
| Recombination endonuclease VII | CDS | 160083 | 160556 | 474 | forward |
| Anaerobic NTP reductase large subunit | CDS | 160553 | 162370 | 1818 | forward |
| Anaerobic NTP reductase small subunit | CDS | 162367 | 162837 | 471 | forward |
| hypothetical protein | CDS | 162815 | 162931 | 117 | forward |
| hypothetical protein | CDS | 162948 | 163160 | 213 | forward |
| hypothetical protein | CDS | 163163 | 163471 | 309 | forward |
| hypothetical protein | CDS | 163638 | 163820 | 183 | forward |
| hypothetical protein | CDS | 163817 | 164065 | 249 | forward |
| hypothetical protein | CDS | 164073 | 164366 | 294 | forward |
| hypothetical protein | CDS | 164374 | 164508 | 135 | forward |
| hypothetical protein | CDS | 164505 | 164705 | 201 | forward |
| hypothetical protein | CDS | 164769 | 165008 | 240 | forward |
| hypothetical protein | CDS | 165075 | 165407 | 333 | forward |
| hypothetical protein | CDS | 165404 | 165631 | 228 | forward |
| hypothetical protein | CDS | 165628 | 165897 | 270 | forward |
| RNA polymerase sigma factor | CDS | 165970 | 166527 | 558 | forward |
| hypothetical protein | CDS | 166517 | 166726 | 210 | forward |
| hypothetical protein | CDS | 166728 | 167051 | 324 | forward |
| hypothetical protein | CDS | 167026 | 167232 | 207 | forward |
| hypothetical protein | CDS | 167272 | 167445 | 174 | forward |

**Table S6**. List of genome features of phage A6

| Name | Type | Start | Stop | Length | Direction |
| --- | --- | --- | --- | --- | --- |
| Putative terminase large subunit | CDS | 1 | 1602 | 1602 | forward |
| Hypothetical protein | CDS | 1619 | 3085 | 1467 | forward |
| Hypothetical protein | CDS | 3085 | 3585 | 501 | forward |
| Hypothetical protein | CDS | 3585 | 3917 | 333 | forward |
| signal peptide peptidase SppA | CDS | 3929 | 5275 | 1347 | forward |
| Structural protein | CDS | 5287 | 5664 | 378 | forward |
| Major capsid protein | CDS | 5698 | 6804 | 1107 | forward |
| Hypothetical protein | CDS | 6826 | 7275 | 450 | forward |
| Hypothetical protein | CDS | 7275 | 7757 | 483 | forward |
| Hypothetical protein | CDS | 7754 | 8155 | 402 | forward |
| Hypothetical protein | CDS | 8130 | 8729 | 600 | forward |
| DUF3383 family protein | CDS | 8730 | 10082 | 1353 | forward |
| DUF3277 family protein | CDS | 10098 | 10544 | 447 | forward |
| Hypothetical protein | CDS | 10618 | 11016 | 399 | forward |
| Hypothetical protein | CDS | 11019 | 11258 | 240 | forward |
| Putative tail tapemeasure protein | CDS | 11258 | 13465 | 2208 | forward |
| Hypothetical protein | CDS | 13465 | 14265 | 801 | forward |
| Hypothetical protein | CDS | 14265 | 14606 | 342 | forward |
| Hypothetical protein | CDS | 14606 | 15583 | 978 | forward |
| Putative baseplate assembly protein | CDS | 15583 | 16206 | 624 | forward |
| Hypothetical protein | CDS | 16206 | 16625 | 420 | forward |
| Baseplate assembly protein | CDS | 16625 | 18094 | 1470 | forward |
| Putative baseplate assembly protein | CDS | 18097 | 18954 | 858 | forward |
| Hypothetical protein | CDS | 18954 | 19256 | 303 | forward |
| Putative tail fiber protein | CDS | 19259 | 20407 | 1149 | forward |
| Putative tail fibers protein | CDS | 20454 | 22793 | 2340 | forward |
| Hypothetical protein | CDS | 22873 | 23067 | 195 | forward |
| Hypothetical protein | CDS | 23068 | 23433 | 366 | forward |
| Putative thymidylate synthase | CDS | 23477 | 24376 | 900 | reverse |
| Dihydrofolate reductase | CDS | 24378 | 24923 | 546 | reverse |
| Hypothetical protein | CDS | 24920 | 25180 | 261 | reverse |
| Hypothetical protein | CDS | 25181 | 25696 | 516 | reverse |
| Hypothetical protein | CDS | 25710 | 26069 | 360 | reverse |
| Helix-turn-helix transcriptional regulator | CDS | 26071 | 26370 | 300 | reverse |
| Hypothetical protein | CDS | 26363 | 26575 | 213 | reverse |
| Hypothetical protein | CDS | 26577 | 26960 | 384 | reverse |
| Hypothetical protein | CDS | 27205 | 27645 | 441 | reverse |
| Hypothetical protein | CDS | 27648 | 28235 | 588 | reverse |
| Hypothetical protein | CDS | 28265 | 28402 | 138 | reverse |
| Hypothetical protein | CDS | 28580 | 28810 | 231 | forward |
| Putative DNA ligase | CDS | 28801 | 29901 | 1101 | reverse |
| Hypothetical protein | CDS | 29898 | 30155 | 258 | reverse |
| Hypothetical protein | CDS | 30115 | 30351 | 237 | reverse |
| Hypothetical protein | CDS | 30365 | 30520 | 156 | reverse |
| Hypothetical protein | CDS | 30539 | 30751 | 213 | reverse |
| Hypothetical protein | CDS | 30748 | 30957 | 210 | reverse |
| DNA polymerase | CDS | 31019 | 31573 | 555 | reverse |
| Hypothetical protein | CDS | 31615 | 31731 | 117 | forward |
| Putative endonuclease | CDS | 31783 | 32268 | 486 | reverse |
| Putative DNA polymerase | CDS | 32336 | 34519 | 2184 | reverse |
| Hypothetical protein | CDS | 34778 | 35200 | 423 | forward |
| Putative minor tail protein | CDS | 35202 | 36002 | 801 | forward |
| Putative deoxynucleotide monophosphate kinase | CDS | 36064 | 36807 | 744 | forward |
| Hypothetical protein | CDS | 36816 | 37016 | 201 | forward |
| Putative DNA primase/helicase | CDS | 37009 | 38994 | 1986 | forward |
| Hypothetical protein | CDS | 38969 | 39250 | 282 | forward |
| Hypothetical protein | CDS | 39247 | 39396 | 150 | forward |
| Hypothetical protein | CDS | 39469 | 40326 | 858 | forward |
| Putative exodeoxyribonuclease | CDS | 40389 | 41435 | 1047 | forward |
| HNH endonuclease | CDS | 41398 | 41913 | 516 | forward |
| Hypothetical protein | CDS | 41910 | 42410 | 501 | forward |
| Hypothetical protein | CDS | 42432 | 42680 | 249 | forward |
| Hypothetical protein | CDS | 42656 | 43411 | 756 | forward |
| Hypothetical protein | CDS | 43392 | 43715 | 324 | forward |
| Hypothetical protein | CDS | 43708 | 44046 | 339 | forward |
| Ribonucleoside-diphosphate reductase subunit alpha | CDS | 44093 | 46327 | 2235 | forward |
| Hypothetical protein | CDS | 46299 | 46640 | 342 | forward |
| Ribonucleotide-diphosphate reductase subunit beta | CDS | 46637 | 47710 | 1074 | forward |
| Putative glutaredoxin | CDS | 47710 | 47952 | 243 | forward |
| Putative membrane protein | CDS | 47945 | 48151 | 207 | forward |
| Putative anaerobic nucleoside diphosphate reductase | CDS | 48200 | 49399 | 1200 | forward |
| Hypothetical protein | CDS | 49424 | 49534 | 111 | forward |
| Putative HNH homing endonuclease | CDS | 49512 | 49976 | 465 | forward |
| Hypothetical protein | CDS | 50017 | 50160 | 144 | reverse |
| Putative ribonucleotide reductase of class III | CDS | 50232 | 51086 | 855 | forward |
| Hypothetical protein | CDS | 51149 | 51484 | 336 | forward |
| Phage membrane protein | CDS | 51487 | 51687 | 201 | forward |
| Hypothetical protein | CDS | 51684 | 52079 | 396 | forward |
| Hypothetical protein | CDS | 52076 | 52375 | 300 | forward |
| Putative anaerobic ribonucleoside-triphosphate reductase activating enzyme | CDS | 52385 | 52870 | 486 | forward |
| Hypothetical protein | CDS | 52833 | 53108 | 276 | forward |
| Hypothetical protein | CDS | 53124 | 53390 | 267 | forward |
| Hypothetical protein | CDS | 53393 | 53713 | 321 | forward |
| Hypothetical protein | CDS | 53768 | 54283 | 516 | forward |
| Hypothetical protein | CDS | 54276 | 54605 | 330 | forward |
| Putative ribose-phosphate pyrophosphokinase | CDS | 54565 | 55446 | 882 | forward |
| HNH endonuclease | CDS | 55455 | 55946 | 492 | forward |
| Putative nicotinamide phosphoribosyltransferase | CDS | 55961 | 57742 | 1782 | forward |
| Hypothetical protein | CDS | 57796 | 58131 | 336 | forward |
| Putative membrane protein | CDS | 58113 | 58289 | 177 | forward |
| Putative rIIA protein | CDS | 58318 | 60684 | 2367 | forward |
| rIIB protein | CDS | 60764 | 61873 | 1110 | forward |
| Hypothetical protein | CDS | 61974 | 62522 | 549 | forward |
| Polynucleotide kinase | CDS | 62500 | 63195 | 696 | forward |
| Hypothetical protein | CDS | 63206 | 63670 | 465 | forward |
| Hypothetical protein | CDS | 63723 | 64070 | 348 | forward |
| Hypothetical protein | CDS | 64036 | 64233 | 198 | forward |
| Hypothetical protein | CDS | 64230 | 64499 | 270 | forward |
| Hypothetical protein | CDS | 64502 | 64828 | 327 | forward |
| Hypothetical protein | CDS | 64803 | 65030 | 228 | forward |
| Hypothetical protein | CDS | 65023 | 65223 | 201 | forward |
| Putative phosphatase | CDS | 65224 | 66009 | 786 | forward |
| Hypothetical protein | CDS | 66096 | 66248 | 153 | forward |
| Hypothetical protein | CDS | 66253 | 66348 | 96 | forward |
| Hypothetical protein | CDS | 66366 | 66464 | 99 | forward |
| Hypothetical protein | CDS | 66520 | 66900 | 381 | forward |
| Hypothetical protein | CDS | 66990 | 67283 | 294 | forward |
| Hypothetical protein | CDS | 67369 | 67881 | 513 | forward |
| Hypothetical protein | CDS | 67974 | 68270 | 297 | forward |
| Hypothetical protein | CDS | 68264 | 68596 | 333 | forward |
| Hypothetical protein | CDS | 68688 | 68960 | 273 | forward |
| Hypothetical protein | CDS | 69046 | 69450 | 405 | forward |
| Hypothetical protein | CDS | 69552 | 69755 | 204 | forward |
| Hypothetical protein | CDS | 69843 | 70385 | 543 | forward |
| Hypothetical protein | CDS | 70452 | 70685 | 234 | forward |
| Hypothetical protein | CDS | 70778 | 71119 | 342 | forward |
| Putative phage protein | CDS | 71194 | 71286 | 93 | reverse |
| Putative phage protein | CDS | 71340 | 71870 | 531 | forward |
| Hypothetical protein | CDS | 71950 | 72198 | 249 | forward |
| Hypothetical protein | CDS | 73241 | 73402 | 162 | forward |
| Hypothetical protein | CDS | 73911 | 74462 | 552 | reverse |
| Hypothetical protein | CDS | 74516 | 74740 | 225 | reverse |
| Hypothetical protein | CDS | 74740 | 74835 | 96 | reverse |
| Putative membrane protein | CDS | 74817 | 75320 | 504 | reverse |
| Hypothetical protein | CDS | 75317 | 75535 | 219 | reverse |
| Hypothetical protein | CDS | 75619 | 76200 | 582 | reverse |
| Hypothetical protein | CDS | 76200 | 76544 | 345 | reverse |
| Hypothetical protein | CDS | 76537 | 76830 | 294 | reverse |
| Hypothetical protein | CDS | 76830 | 77219 | 390 | reverse |
| Hypothetical protein | CDS | 77212 | 77610 | 399 | reverse |
| Putative lysozyme | CDS | 77662 | 78126 | 465 | reverse |
| Putative tail protein | CDS | 78126 | 79010 | 885 | reverse |
| Hypothetical protein | CDS | 79007 | 79378 | 372 | reverse |
| Hypothetical protein | CDS | 79426 | 80016 | 591 | reverse |
| Hypothetical protein | CDS | 80010 | 80243 | 234 | reverse |
| Hypothetical protein | CDS | 80302 | 80430 | 129 | forward |
| Hypothetical protein | CDS | 80636 | 81007 | 372 | forward |
| Putative vWFA protein | CDS | 81089 | 82504 | 1416 | forward |
| Hypothetical protein | CDS | 82511 | 82624 | 114 | reverse |
| tRNA-Pro | tRNA | 82670 | 82744 | 75 | forward |
| Hypothetical protein | CDS | 82749 | 82964 | 216 | reverse |
| tRNA-Asn | tRNA | 83078 | 83152 | 75 | forward |
| tRNA-Tyr | tRNA | 83163 | 83248 | 86 | forward |
| tRNA-Asp | tRNA | 83256 | 83330 | 75 | forward |
| Hypothetical protein | CDS | 83336 | 83509 | 174 | forward |
| Hypothetical protein | CDS | 83580 | 83681 | 102 | forward |
| tRNA-Lys | tRNA | 83763 | 83836 | 74 | forward |
| tRNA-Met | tRNA | 83843 | 83917 | 75 | forward |
| tRNA-IIe | tRNA | 83922 | 83994 | 73 | forward |
| Hypothetical protein | CDS | 84066 | 84158 | 93 | reverse |
| tRNA-Arg | tRNA | 84244 | 84318 | 75 | forward |
| Hypothetical protein | CDS | 84336 | 84677 | 342 | forward |
| Hypothetical protein | CDS | 85004 | 85141 | 138 | forward |
| tRNA-Leu | tRNA | 85229 | 85304 | 76 | forward |
| tRNA-Lys | tRNA | 85314 | 85387 | 74 | forward |
| tRNA-Ala | tRNA | 85396 | 85469 | 74 | forward |
| tRNA-Gly | tRNA | 85478 | 85550 | 73 | forward |
| tRNA-Thr | tRNA | 85559 | 85633 | 75 | forward |
| tRNA-Val | tRNA | 85731 | 85803 | 73 | forward |
| tRNA-Leu | tRNA | 85807 | 85882 | 76 | forward |
| tRNA-Arg | tRNA | 85890 | 85963 | 74 | forward |
| Hypothetical protein | CDS | 85985 | 86542 | 558 | forward |
| tRNA-Gln | tRNA | 86552 | 86624 | 73 | forward |
| tRNA-Leu | tRNA | 86629 | 86705 | 77 | forward |
| tRNA-Gln | tRNA | 86713 | 86786 | 74 | forward |
| tRNA-His | tRNA | 86820 | 86893 | 74 | forward |
| tRNA-Phe | tRNA | 86902 | 86975 | 74 | forward |
| Putative membrane protein | CDS | 86994 | 87728 | 735 | forward |
| tRNA-Ser | tRNA | 87730 | 87820 | 91 | forward |
| tRNA-Cys | tRNA | 87827 | 87900 | 74 | forward |
| Hypothetical protein | CDS | 87938 | 88126 | 189 | forward |
| Hypothetical protein | CDS | 88126 | 88326 | 201 | forward |

**Table S7.** List of genome features of phage A7

| Name | Type | Start | Stop | Length | Direction |
| --- | --- | --- | --- | --- | --- |
| Putative exonuclease | CDS | 2 | 412 | 411 | forward |
| Putative DUF5516 domain containing protein | CDS | 400 | 513 | 114 | forward |
| DUF2717 domain-containing protein | CDS | 611 | 865 | 255 | forward |
| DUF5476 family protein | CDS | 870 | 1136 | 267 | forward |
| Hypothetical protein LNA7_001 | CDS | 1136 | 1537 | 402 | forward |
| Hypothetical protein | CDS | 1541 | 1843 | 303 | forward |
| Hypothetical protein | CDS | 1858 | 2250 | 393 | forward |
| Putative head-to-tail connector | CDS | 2250 | 3860 | 1611 | forward |
| Putative capsid and scaffold protein | CDS | 3960 | 4883 | 924 | forward |
| Putative capsid protein | CDS | 4982 | 6019 | 1038 | forward |
| Putative minor capsid protein | CDS | 6028 | 6177 | 150 | forward |
| Putative tail tubular protein A | CDS | 6246 | 6836 | 591 | forward |
| Putative tail tubular protein B | CDS | 6857 | 9241 | 2385 | forward |
| DUF2833 domain-containing protein | CDS | 9322 | 9738 | 417 | forward |
| Putative internal virion protein B | CDS | 9743 | 10333 | 591 | forward |
| Putative internal virion protein C | CDS | 10340 | 12583 | 2244 | forward |
| Putative internal virion protein D | CDS | 12610 | 16566 | 3957 | forward |
| Putative tail fiber protein | CDS | 16639 | 18438 | 1800 | forward |
| Putative holin | CDS | 18467 | 18670 | 204 | forward |
| Terminase small subunit | CDS | 18676 | 18945 | 270 | forward |
| Putative endopeptidase Rz | CDS | 19040 | 19483 | 444 | forward |
| Putative terminase large subunit | CDS | 19493 | 21253 | 1761 | forward |
| Hypothetical protein | CDS | 21291 | 21731 | 441 | forward |
| Hypothetical protein | CDS | 21897 | 22046 | 150 | forward |
| Hypothetical protein | CDS | 22761 | 22895 | 135 | reverse |
| Hypothetical protein | CDS | 22924 | 23055 | 132 | reverse |
| Putative receptor binding protein kinase | CDS | 23231 | 23596 | 366 | forward |
| Hypothetical protein | CDS | 23596 | 23733 | 138 | forward |
| Hypothetical protein | CDS | 23767 | 23937 | 171 | forward |
| Hypothetical protein | CDS | 23937 | 24071 | 135 | forward |
| Putative protein kinase | CDS | 24108 | 25169 | 1062 | forward |
| Putative RNA polymerase | CDS | 25240 | 27891 | 2652 | forward |
| Hypothetical protein | CDS | 28080 | 28208 | 129 | forward |
| Putative dGTP triphosphohydrolase inhibitor | CDS | 28210 | 28467 | 258 | forward |
| Putative DNA ligase | CDS | 28546 | 29568 | 1023 | forward |
| Hypothetical protein | CDS | 29636 | 29725 | 90 | forward |
| Hypothetical protein | CDS | 29751 | 30011 | 261 | forward |
| Putative nucleotide kinase | CDS | 30011 | 30487 | 477 | forward |
| Hypothetical protein | CDS | 30477 | 30599 | 123 | forward |
| Putative host RNA polymerase inhibitor | CDS | 30601 | 30795 | 195 | forward |
| Putative ssDNA-binding protein | CDS | 30861 | 31559 | 699 | forward |
| Putative endonuclease | CDS | 31560 | 32015 | 456 | forward |
| Putative lysin | CDS | 32015 | 32470 | 456 | forward |
| Putative DNA primase/helicase | CDS | 32542 | 34245 | 1704 | forward |
| Putative HNH endonuclease | CDS | 34257 | 34718 | 462 | forward |
| Hypothetical protein | CDS | 34706 | 34918 | 213 | forward |
| Hypothetical protein | CDS | 34938 | 35207 | 270 | forward |
| Hypothetical protein | CDS | 35152 | 35694 | 543 | forward |
| Putative DNA polymerase | CDS | 35713 | 37722 | 2010 | forward |
| Putative HNH homing endonuclease | CDS | 37758 | 38153 | 396 | forward |
| DNA polymerase | CDS | 38316 | 38429 | 114 | forward |
| Putative protein suppressor of silencing | CDS | 38449 | 38748 | 300 | forward |
| Hypothetical protein | CDS | 38748 | 38957 | 210 | forward |
| Host recBCD nuclease inhibitor | CDS | 38957 | 39115 | 159 | forward |
| Putative exonuclease | CDS | 39102 | 39590 | 489 | forward |

**Table S8.** List of genome features of phage A8

| Name | Type | Start | Stop | Length | Direction |
| --- | --- | --- | --- | --- | --- |
| Putative DNA polymerase | CDS | 1 | 264 | 264 | reverse |
| Putative DNA helicase | CDS | 200 | 1693 | 1494 | reverse |
| Putative DNA primase | CDS | 1608 | 2513 | 906 | reverse |
| Hypothetical protein | CDS | 2739 | 3458 | 720 | reverse |
| Hypothetical protein | CDS | 3458 | 3919 | 462 | reverse |
| Hypothetical protein | CDS | 3951 | 4091 | 141 | reverse |
| Hypothetical protein | CDS | 4860 | 5402 | 543 | reverse |
| Putative protein kinase | CDS | 5926 | 6612 | 687 | reverse |
| Hypothetical protein | CDS | 7606 | 8001 | 396 | reverse |
| Hypothetical protein | CDS | 7998 | 8186 | 189 | reverse |
| Hypothetical protein | CDS | 8189 | 8680 | 492 | reverse |
| Hypothetical protein | CDS | 8989 | 9330 | 342 | forward |
| Hypothetical protein | CDS | 9883 | 10509 | 627 | reverse |
| Putative phage membrane protein | CDS | 10570 | 10917 | 348 | reverse |
| Putative lysozyme | CDS | 10907 | 11389 | 483 | reverse |
| Putative Ig-like domain-containing protein | CDS | 11400 | 11885 | 486 | reverse |
| Putative terminase large subunit | CDS | 11889 | 13640 | 1752 | reverse |
| Hypothetical protein | CDS | 13640 | 13870 | 231 | reverse |
| Putative pin holin | CDS | 13920 | 14126 | 207 | reverse |
| Putative tail fibre protein | CDS | 14137 | 16251 | 2115 | reverse |
| Putative internal virion protein | CDS | 16317 | 20072 | 3756 | reverse |
| Hypothetical protein | CDS | 20102 | 22366 | 2265 | reverse |
| Hypothetical protein | CDS | 22374 | 23087 | 714 | reverse |
| Putative tail tubular protein B | CDS | 23090 | 25654 | 2565 | reverse |
| Putative tail tubular protein A | CDS | 25658 | 26254 | 597 | reverse |
| Putative major capsid protein | CDS | 26320 | 27309 | 990 | reverse |
| Hypothetical protein | CDS | 27371 | 28117 | 747 | reverse |
| Putative head-to-tail joining protein | CDS | 28114 | 29634 | 1521 | reverse |
| Hypothetical protein | CDS | 29643 | 30068 | 426 | reverse |
| Hypothetical protein | CDS | 30065 | 30580 | 516 | reverse |
| Putative DNA-directed RNA polymerase | CDS | 30923 | 33322 | 2400 | reverse |
| Hypothetical protein | CDS | 33319 | 33633 | 315 | reverse |
| Putative DNA ligase | CDS | 33630 | 34520 | 891 | reverse |
| Hypothetical protein | CDS | 34755 | 35258 | 504 | reverse |
| Putative ribonuclease H-like domain containing protein | CDS | 35251 | 36030 | 780 | reverse |
| Putative DNA endonuclease | CDS | 36027 | 36407 | 381 | reverse |
| Putative phage exonuclease | CDS | 36380 | 37318 | 939 | reverse |
| Hypothetical protein | CDS | 37366 | 38232 | 867 | reverse |
| Putative DNA polymerase | CDS | 38229 | 40103 | 1875 | reverse |

**Table S9.** List of genome features of phage A9

| Name | Type | Start | Stop | Length | Direction |
| --- | --- | --- | --- | --- | --- |
| Hypothetical protein | CDS | 764 | 1006 | 243 | reverse |
| Hypothetical protein | CDS | 1216 | 1320 | 105 | reverse |
| Holin | CDS | 1313 | 1666 | 354 | reverse |
| Putative transclycosylase | CDS | 1663 | 2472 | 810 | reverse |
| Hypothetical protein | CDS | 2473 | 2565 | 93 | reverse |
| Hypothetical protein | CDS | 2550 | 2825 | 276 | reverse |
| Putative DNA delivery | CDS | 2836 | 3459 | 624 | reverse |
| Hypothetical protein | CDS | 3461 | 3571 | 111 | reverse |
| Minor capsid protein | CDS | 3574 | 3828 | 255 | reverse |
| P34 virion membrane protein | CDS | 3838 | 4044 | 207 | reverse |
| DNA delivery protein | CDS | 4057 | 4221 | 165 | reverse |
| Hypothetical protein | CDS | 4493 | 4615 | 123 | reverse |
| DNA packaging | CDS | 4715 | 4858 | 144 | reverse |
| Putative major capsid protein | CDS | 4876 | 5295 | 420 | reverse |
| Putative major capsid protein | CDS | 5337 | 6062 | 726 | reverse |
| DNA packaging | CDS | 6069 | 6197 | 129 | reverse |
| Hypothetical protein | CDS | 6197 | 6325 | 129 | reverse |
| Putative DNA packaging ATPase | CDS | 6411 | 7019 | 609 | reverse |
| Putative assembly | CDS | 7030 | 7338 | 309 | reverse |
| Putative minor capsid protein | CDS | 7363 | 7605 | 243 | reverse |
| Putative minor capsid protein | CDS | 7605 | 7718 | 114 | reverse |
| Putative assembly protein | CDS | 7866 | 8072 | 207 | reverse |
| Assembly protein | CDS | 8062 | 8322 | 261 | reverse |
| Spike protein | CDS | 8341 | 8583 | 243 | reverse |
| Spike protein | CDS | 8664 | 9362 | 699 | reverse |
| Putative penton | CDS | 9362 | 9616 | 255 | reverse |
| Putative penton | CDS | 9613 | 9741 | 129 | reverse |
| Putative receptor binding protein | CDS | 9745 | 9858 | 114 | reverse |
| Putative receptor binding protein | CDS | 9989 | 10261 | 273 | reverse |
| Putative receptor binding protein | CDS | 10228 | 10449 | 222 | reverse |
| Putative receptor binding protein | CDS | 10488 | 10814 | 327 | reverse |
| Receptor binding protein | CDS | 10915 | 11025 | 111 | reverse |
| Putative receptor binding protein | CDS | 11087 | 11494 | 408 | reverse |
| Putative muramidase | CDS | 11494 | 11988 | 495 | reverse |
| Putative DNA polymerase | CDS | 11945 | 12937 | 993 | reverse |
| Putative DNA polymerase | CDS | 12957 | 13325 | 369 | reverse |
| Putative DNA polymerase | CDS | 13374 | 13598 | 225 | reverse |
| Putative terminal protein | CDS | 13602 | 14342 | 741 | reverse |

**Table S10.** List of genome features of phage A10

| Name | Type | Start | Stop | Length | Direction |
| --- | --- | --- | --- | --- | --- |
| Hypothetical protein | CDS | 1 | 1350 | 1350 | reverse |
| Hypothetical protein | CDS | 1416 | 5171 | 3756 | reverse |
| Hypothetical protein | CDS | 5201 | 7465 | 2265 | reverse |
| Hypothetical protein | CDS | 7473 | 8186 | 714 | reverse |
| Putative tail tubular protein B | CDS | 8189 | 10747 | 2559 | reverse |
| Putative tail tubular protein A | CDS | 10751 | 11347 | 597 | reverse |
| Putative capsid and scaffold protein | CDS | 11413 | 12402 | 990 | reverse |
| Hypothetical protein | CDS | 12464 | 13216 | 753 | reverse |
| Putative head-to-tail connector protein | CDS | 13213 | 14733 | 1521 | reverse |
| Hypothetical protein | CDS | 15167 | 15625 | 459 | reverse |
| Putative RNA polymerase | CDS | 16024 | 18423 | 2400 | reverse |
| Hypothetical protein | CDS | 18420 | 18734 | 315 | reverse |
| Putative DNA ligase | CDS | 18731 | 19621 | 891 | reverse |
| Hypothetical protein | CDS | 19856 | 20461 | 606 | reverse |
| Putative ribonuclease H-like domain containing protein | CDS | 20352 | 21131 | 780 | reverse |
| Putative endonuclease | CDS | 21128 | 21508 | 381 | reverse |
| Putative exonuclease | CDS | 21481 | 22419 | 939 | reverse |
| Hypothetical protein | CDS | 22467 | 23279 | 813 | reverse |
| Putative DNA polymerase | CDS | 23330 | 25618 | 2289 | reverse |
| Putative DNA helicase | CDS | 25872 | 27386 | 1515 | reverse |
| Putative DNA primase/helicase | CDS | 27211 | 28116 | 906 | reverse |
| Hypothetical protein | CDS | 28342 | 29061 | 720 | reverse |
| Hypothetical protein | CDS | 29061 | 29522 | 462 | reverse |
| Hypothetical protein | CDS | 29554 | 29694 | 141 | reverse |
| Putative protein kinase | CDS | 30672 | 31724 | 1053 | reverse |
| Hypothetical protein | CDS | 34956 | 35582 | 627 | reverse |
| Putative phage membrane protein | CDS | 35643 | 35990 | 348 | reverse |
| Putative lysozyme | CDS | 35980 | 36462 | 483 | reverse |
| Putative Ig-like domain containing protein | CDS | 36473 | 36958 | 486 | reverse |
| Putative terminase large subunit | CDS | 36962 | 38713 | 1752 | reverse |
| Hypothetical protein | CDS | 38713 | 38943 | 231 | reverse |
| Putative pin holin | CDS | 38993 | 39199 | 207 | reverse |
| Hypothetical protein | CDS | 39210 | 39926 | 717 | reverse |

**Table S11.** List of genome features of phage A11

| Name | Type | Start | Stop | Length | Direction |
| --- | --- | --- | --- | --- | --- |
| Putative internal virion protein A | CDS | 42 | 476 | 435 | reverse |
| Putative tail tubular protein B | CDS | 535 | 2961 | 2427 | reverse |
| Putative capsid and scaffold protein | CDS | 2761 | 4671 | 1911 | forward |
| Putative capsid and scaffold protein | CDS | 4765 | 5640 | 876 | reverse |
| Putative head-tail connector protein | CDS | 5709 | 7340 | 1632 | reverse |
| Putative tail assembly protein | CDS | 7354 | 7659 | 306 | reverse |
| Hypothetical protein | CDS | 7631 | 7993 | 363 | reverse |
| Hypothetical protein | CDS | 8071 | 8346 | 276 | reverse |
| Hypothetical protein | CDS | 8349 | 8591 | 243 | reverse |
| Putative exonuclease | CDS | 8660 | 9559 | 900 | reverse |
| Hypothetical protein | CDS | 9556 | 9765 | 210 | reverse |
| Hypothetical protein | CDS | 9758 | 10141 | 384 | reverse |
| Putative DNA polymerase | CDS | 10152 | 12299 | 2148 | reverse |
| Hypothetical protein | CDS | 12309 | 12779 | 471 | reverse |
| Hypothetical protein | CDS | 12842 | 13045 | 204 | reverse |
| Putative primase/helicase protein | CDS | 13063 | 14754 | 1692 | reverse |
| Hypothetical protein | CDS | 14741 | 15295 | 555 | reverse |
| Putative lysozyme | CDS | 15365 | 15805 | 441 | reverse |
| Endonuclease I | CDS | 15808 | 16251 | 444 | reverse |
| Putative single stranded DNA binding protein | CDS | 16251 | 16955 | 705 | reverse |
| Hypothetical protein | CDS | 17009 | 17374 | 366 | reverse |
| Putative host RNA polymerase inhibitor | CDS | 17371 | 17538 | 168 | reverse |
| Hypothetical protein | CDS | 17535 | 18188 | 654 | reverse |
| Hypothetical protein | CDS | 18185 | 18442 | 258 | reverse |
| Putative DNA ligase | CDS | 18634 | 19776 | 1143 | reverse |
| Hypothetical protein | CDS | 19788 | 20174 | 387 | reverse |
| Hypothetical protein | CDS | 20174 | 20410 | 237 | reverse |
| Hypothetical protein | CDS | 20437 | 20574 | 138 | reverse |
| Putative T3/T7-like RNA polymerase | CDS | 20592 | 23249 | 2658 | reverse |
| Hypothetical protein | CDS | 23378 | 23821 | 444 | reverse |
| Hypothetical protein | CDS | 23823 | 23969 | 147 | reverse |
| Hypothetical protein | CDS | 24020 | 24724 | 705 | reverse |
| Hypothetical protein | CDS | 24721 | 25287 | 567 | reverse |
| Hypothetical protein | CDS | 25302 | 25784 | 483 | reverse |
| Hypothetical protein | CDS | 25877 | 26149 | 273 | reverse |
| Hypothetical protein | CDS | 26151 | 26444 | 294 | reverse |
| Hypothetical protein | CDS | 26441 | 26692 | 252 | reverse |
| Hypothetical protein | CDS | 28084 | 28257 | 174 | reverse |
| Putative terminase large subunit | CDS | 28439 | 30184 | 1746 | reverse |
| Putative Rz-like lysis protein | CDS | 30184 | 30633 | 450 | reverse |
| Putative terminase small subunit | CDS | 30633 | 30890 | 258 | reverse |
| Type II holin | CDS | 30887 | 31102 | 216 | reverse |
| Hypothetical protein | CDS | 31102 | 31458 | 357 | reverse |
| Putative tail fiber protein | CDS | 31498 | 33315 | 1818 | reverse |
| Putative internal virion protein D | CDS | 33378 | 37553 | 4176 | reverse |
| Putative internal virion protein C | CDS | 37566 | 39782 | 2217 | reverse |
| Putative internal virion protein B | CDS | 39775 | 40329 | 555 | reverse |

**Table S12**. BLAST whole genome pairwise comparisons of *E. coli* K12 phages and *P. putida* KT2440 phages. Query cover represent the percentage of query sequence aligned to the target sequence. The similarity percentage describes the similarity between two sequences. (N.D represents no similarity observed between the two target sequences.)

| Phage comparisons | Query cover (%) | Similarity percentage (%) |
| --- | --- | --- |
| *E. coli* K12 phages | | |
| A1 and A2 | N.D | N.D |
| A1 and A6 | 93 | 97.60 |
| A1 and A7 | N.D | N.D |
| A2 and A6 | N.D | N.D |
| A2 and A7 | N.D | N.D |
| A6 and A7 | N.D | N.D |
| *P. putida* KT2440 phages | | |
| A8 and A9 | N.D | N.D |
| A8 and A10 | 97 | 93.72 |
| A8 and A11 | N.D | N.D |
| A9 and A10 | N.D | N.D |
| A9 and A11 | N.D | N.D |
| A10 and A11 | N.D | N.D |

**Table S13.** Host range of phage determined via spot test assay. (-) indicate no lysis spot. (**+**) indicate turbid lysis spot. (**++**) indicate clear lysis spot. (*) indicate the original bacteria host where the phage was isolated from.

| Phage | Bacteria host | | | | | | | |
| --- | --- | --- | --- | --- | --- | --- | --- | --- |
|  | *E. coli* K12 MG1655 | | | | *P. putida* KT2440 | | | |
|  | WT | RP4 plasmid | pMS6198A plasmid | pKJK5 plasmid | WT | RP4 plasmid | pMS6198A plasmid | pKJK5 plasmid |
| Phage A1 | **++** | **++*** | **++** | **++** | **-** | **-** | **-** | **-** |
| Phage A2 | **++** | **++** | **++*** | **++** | **-** | **-** | **-** | **-** |
| Phage A6 | **++*** | **++** | **++** | **++** | **-** | **-** | **-** | **-** |
| Phage A7 | **+** | **+** | **++** | **++*** | **-** | **-** | **-** | **-** |
| Phage A8 | **-** | **-** | **-** | **-** | **++*** | **++** | **++** | **++** |
| Phage A9 | **-** | **++** | **-** | **++** | **-** | **++*** | **-** | **++** |
| Phage A10 | **-** | **-** | **-** | **-** | **++** | **++** | **++*** | **++** |
| Phage A11 | **-** | **-** | **-** | **-** | **++** | **++** | **++** | **++*** |

**
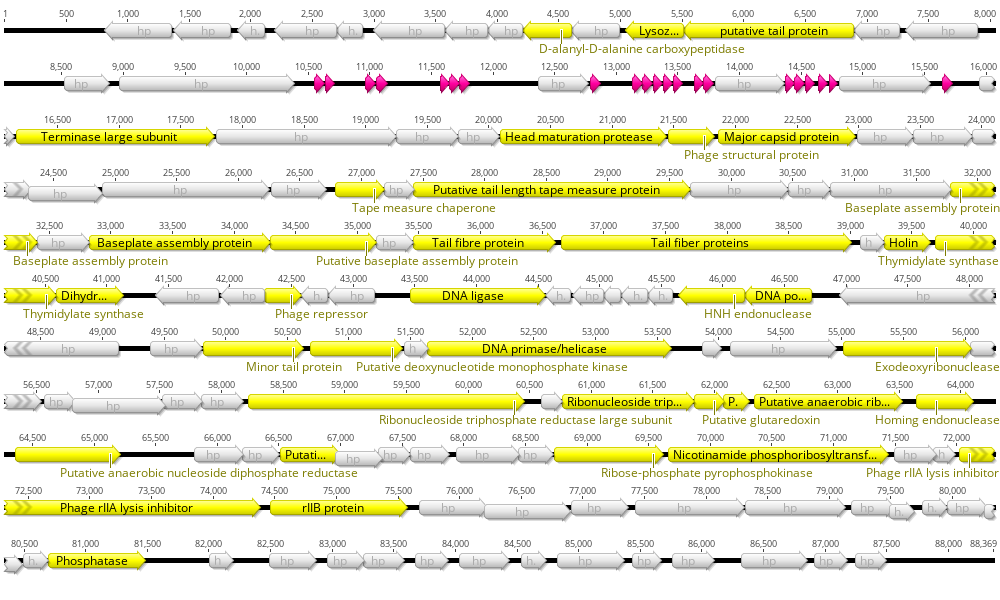
Figure S1.** Genome feature of phage vB_EcoM_LNA1.Yellow arrows represent ORFs with putative functions, grey arrows represent ORFs with hypothetical / unknown function, red arrows represent tRNAs identified.


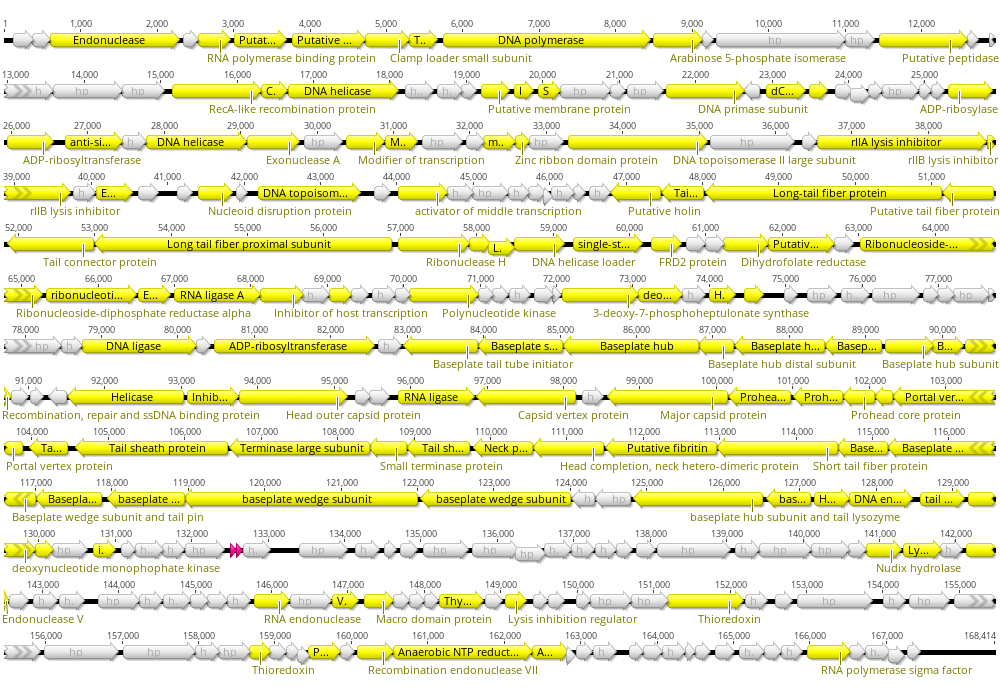
**Figure S2.** Genome feature of phage vB_EcoM_LNA2.Yellow arrows represent ORFs with putative functions, grey arrows represent ORFs with hypothetical / unknown function, red arrows represent tRNAs identified.


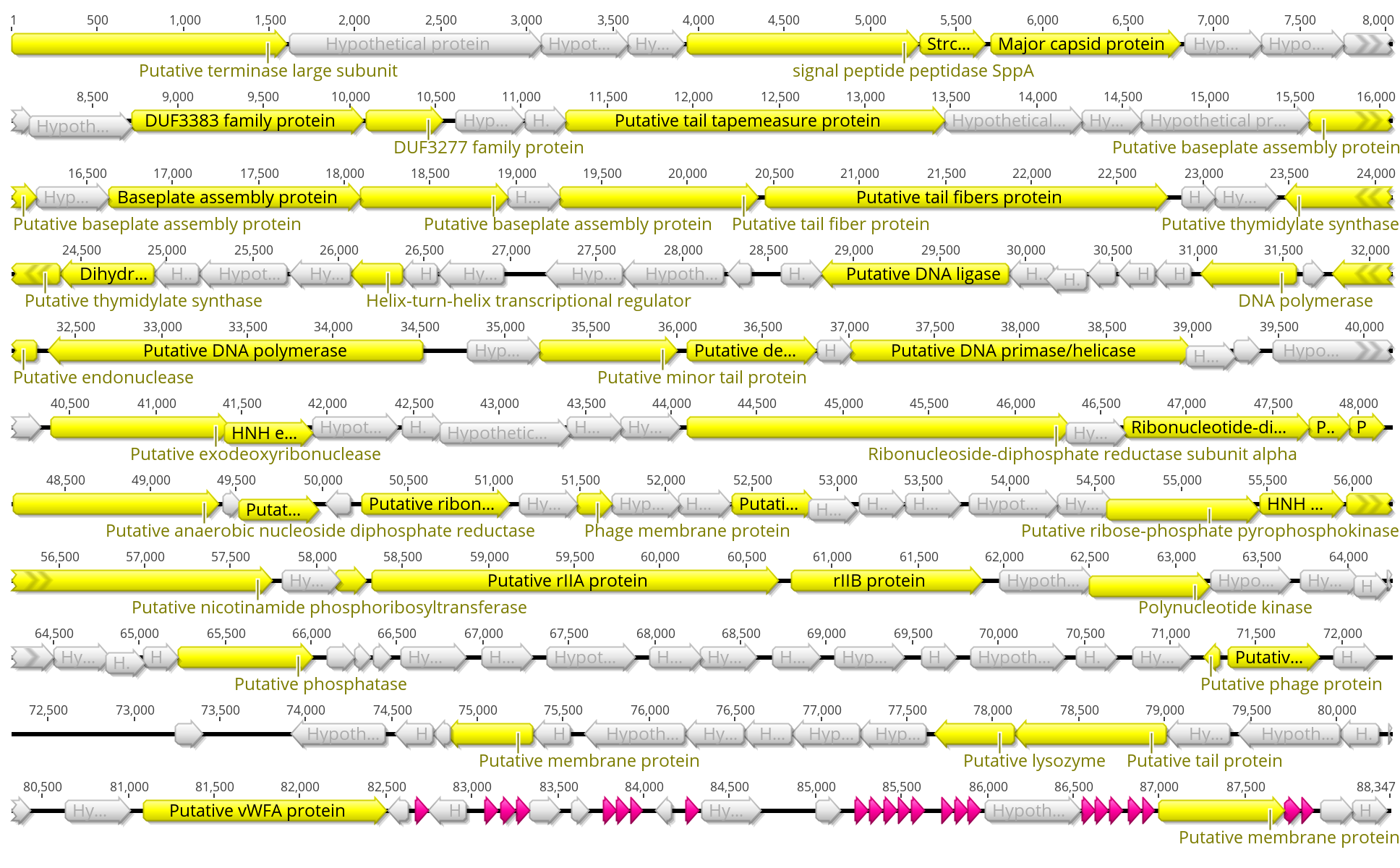


**Figure S3.** Genome feature of phage vB_EcoM_LNA6.Yellow arrows represent ORFs with putative functions, grey arrows represent ORFs with hypothetical / unknown function, red arrows represent tRNAs identified.


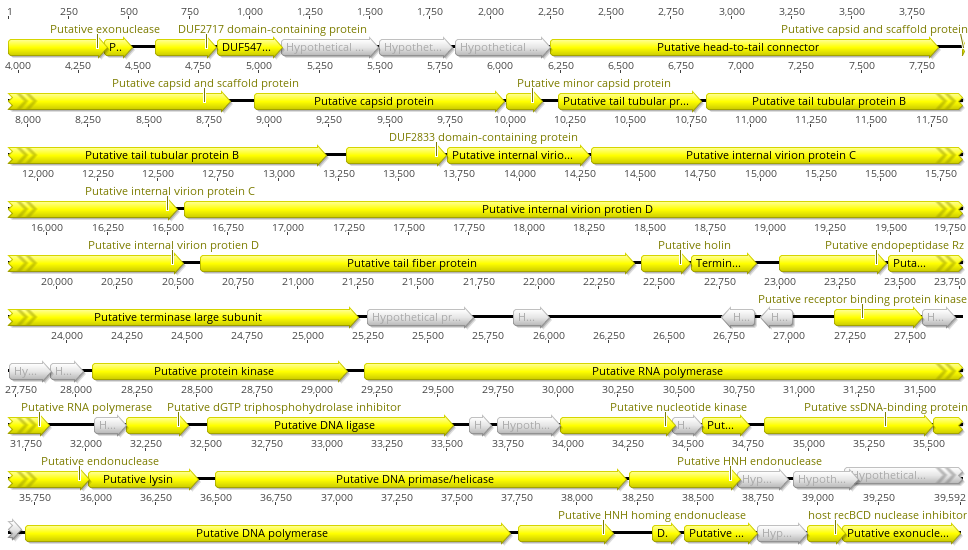


**Figure S4.** Genome feature of phage vB_EcoP_LNA7.Yellow arrows represent ORFs with putative functions, grey arrows represent ORFs with hypothetical / unknown function.


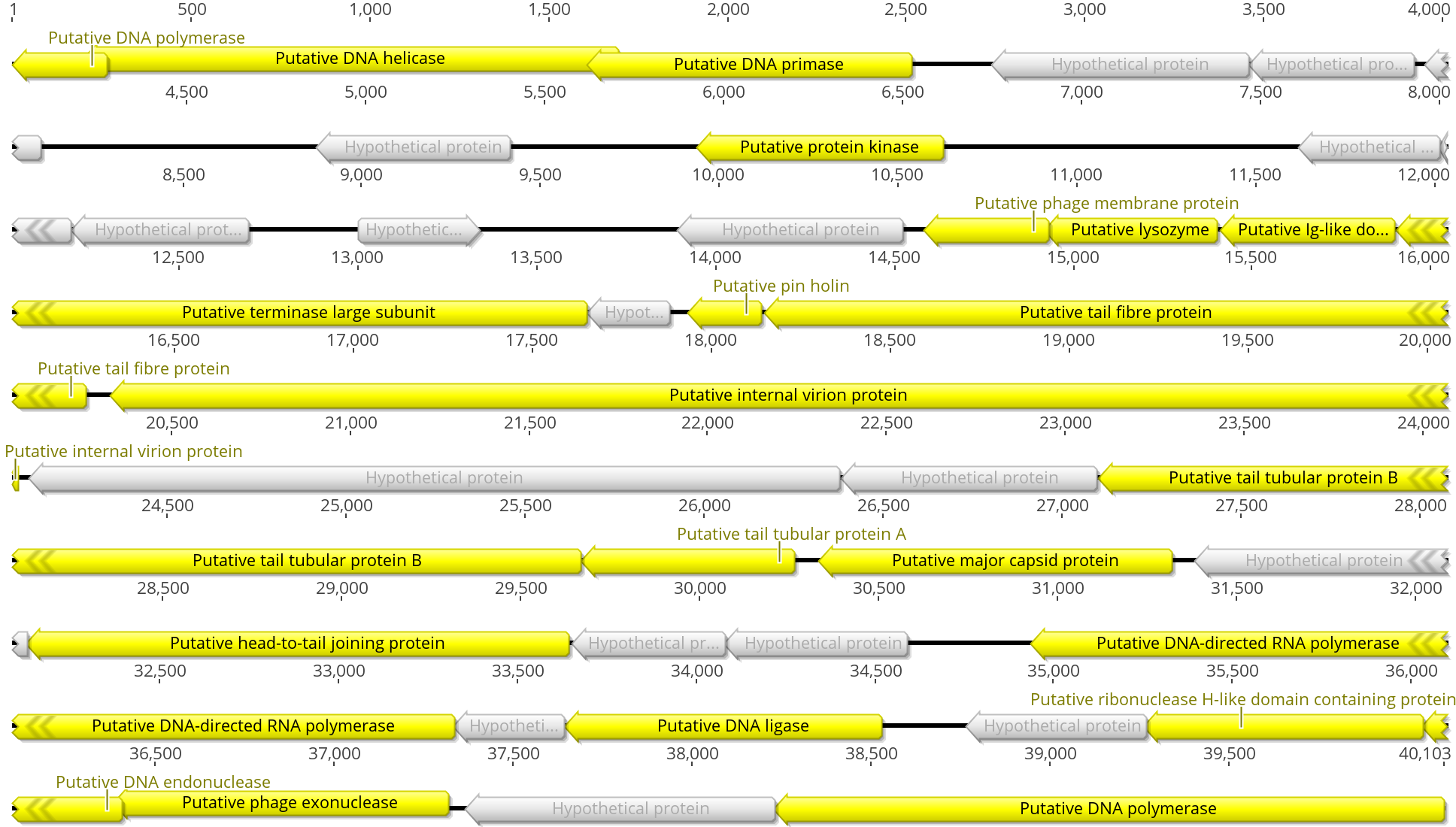


**Figure S5.** Genome feature of phage vB_PputP_LNA8.Yellow arrows represent ORFs with putative functions, grey arrows represent ORFs with hypothetical / unknown function.


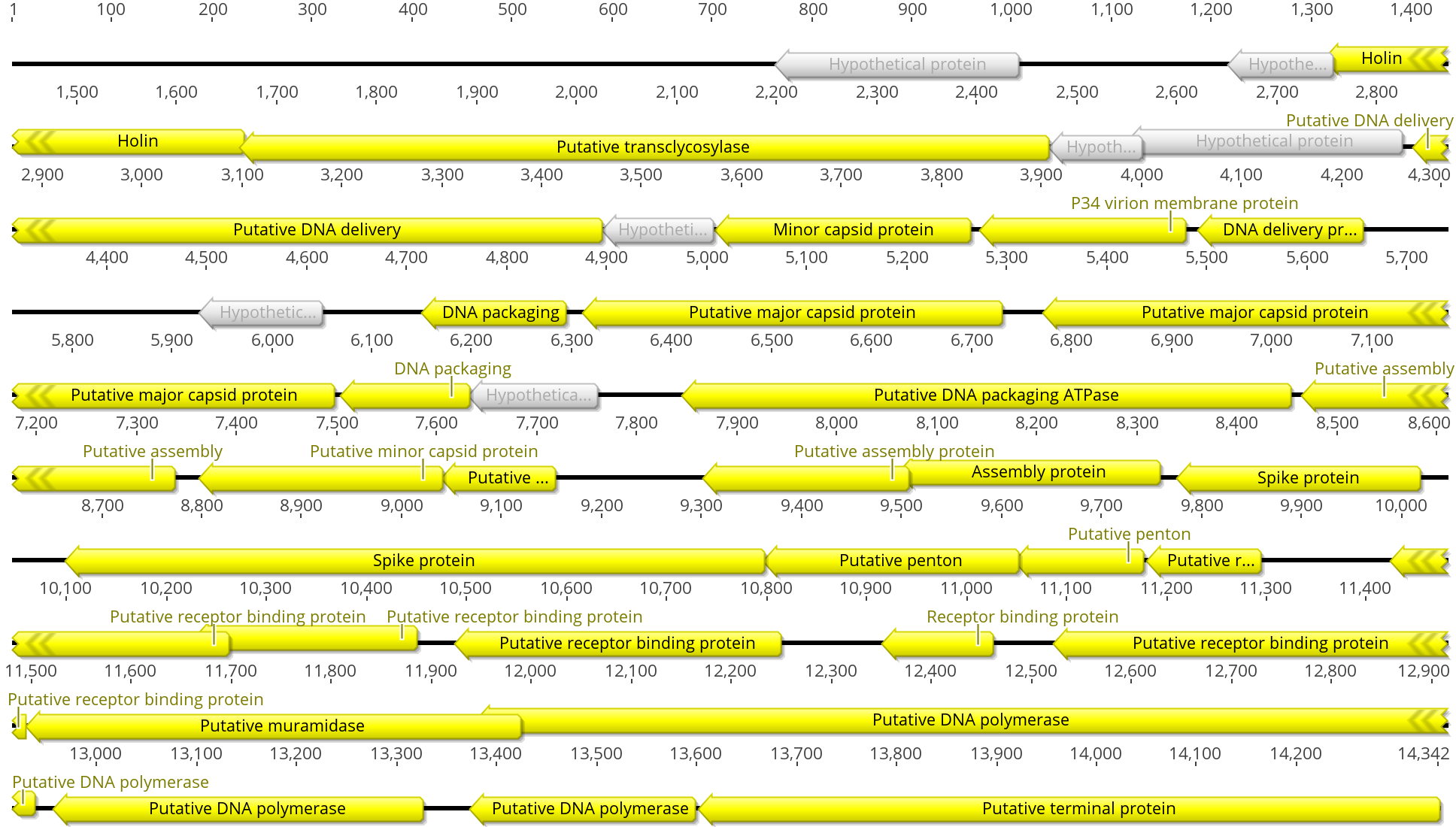


**Figure S6.** Genome feature of phage vB_PputP_LNA9.Yellow arrows represent ORFs with putative functions, grey arrows represent ORFs with hypothetical / unknown function.

**
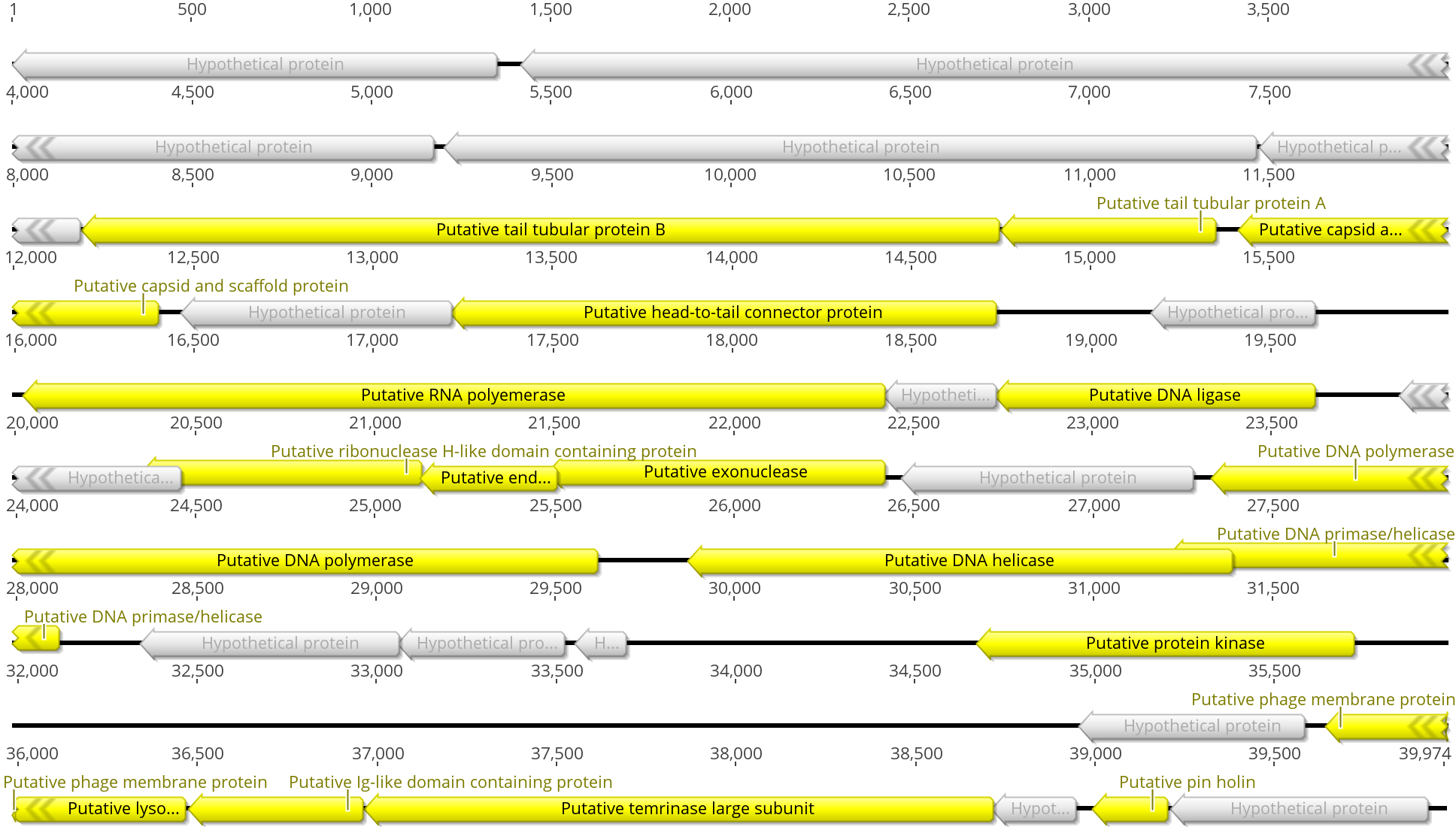
**

**Figure S7.** Genome feature of phage vB_PputP_LNA10.Yellow arrows represent ORFs with putative functions, grey arrows represent ORFs with hypothetical / unknown function.


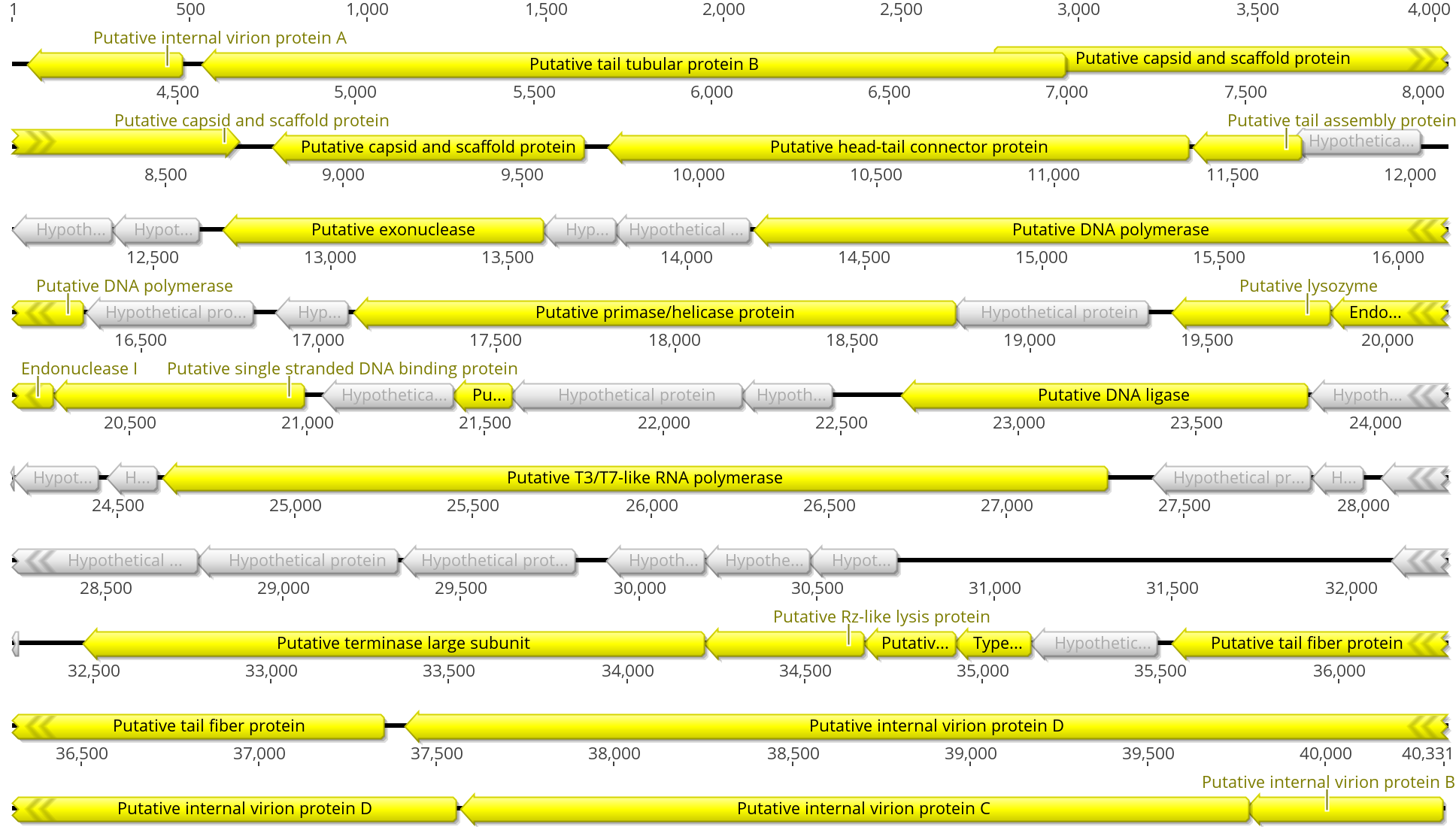


**Figure S8.** Genome feature of phage vB_PputP_LNA11.Yellow arrows represent ORFs with putative functions, grey arrows represent ORFs with hypothetical / unknown function.

*E.coli* K12 MG1655 w/ plasmid RP4


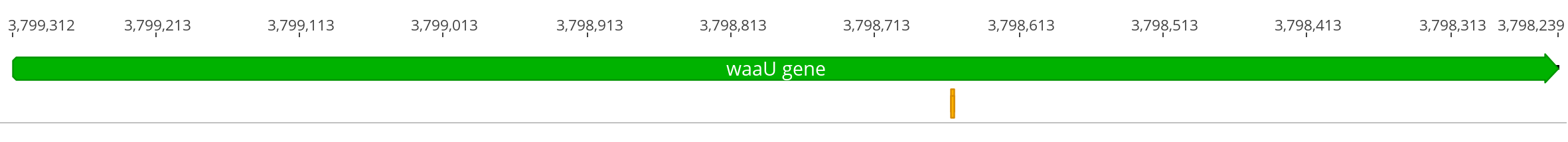


*E.coli* K12 MG1655 w/ plasmid RP4

mutant (Phage A1)

**… AAT CCA TTA GGT GCA AAA …**

**… AAT CCA TTA GGT GC- AAA …**

# Figure S9. Genomic variant comparison of *waaU* gene of *E. coli* K12 MG1655 w/ RP4 plasmid host before and after infection by phage A1. Yellow bar denotes the mutational change observed at nucleotide level.

*E.coli* K12 MG1655 WT

*E.coli* K12 MG1655 WT

mutant (Phage A2)

**… GGC GTA ACT AAC AAC GGT …**

**… GGC GTA ATT AAC AAC GGT …**


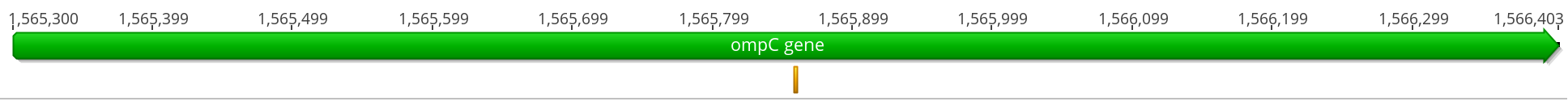


# Figure S10. Genomic variant comparison of *ompC* gene of *E. coli* K12 MG1655 WT before and after infection by phage A2. Yellow bar denotes the mutational change observed at nucleotide level.


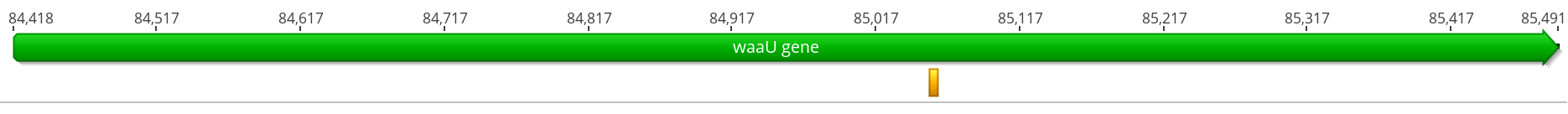


*E.coli* K12 MG1655 WT

*E.coli* K12 MG1655 WT

mutant (Phage A6)

**..ATA GTC ATT ATT AAT CCA TTA GGT..**

**..ATA GTC ATT ATG GTA ATG ACT CCA TTA GGT..**

# Figure S11. Genomic variant comparison of *waaU* gene of *E. coli* K12 MG1655 WT before and after infection by phage A6. Yellow bar denotes the mutational change observed at nucleotide level.

*E.coli* K12 MG1655 w/ plasmid pKJK5

*E.coli* K12 MG1655 w/ plasmid pKJK5

mutant (Phage A7)

**… GAC GAT GCA GCG CGC …**

**… GAC GAT GCA - - G CGC …**


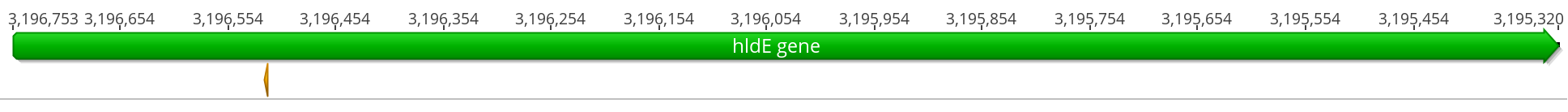


# Figure S12. Genomic variant comparison of *hldE* gene of *E. coli* K12 MG1655 w/ plasmid pKJK5 before and after infection by phage A7. Yellow bar denotes the mutational change observed at nucleotide level.

**B**

**A**


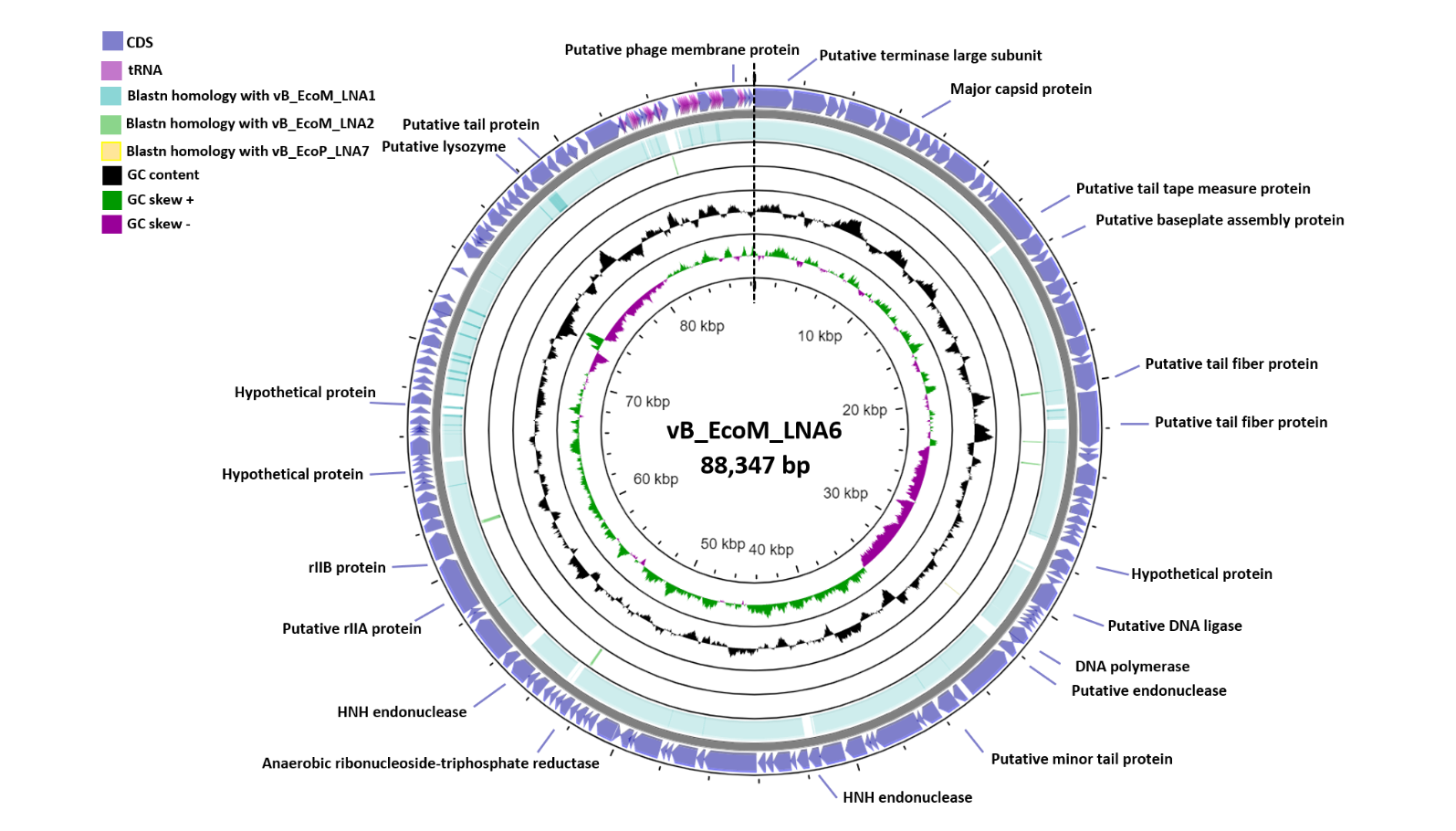


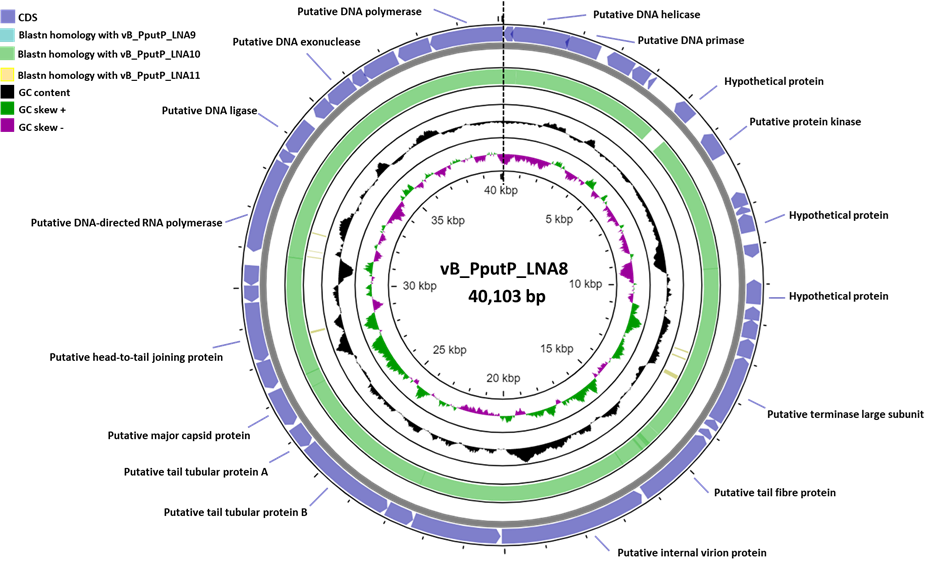


**Figure S13.** Visualisation of whole-genome feature and comparison of *E. coli* K12 phage and *P. putida* phages **A.** Visualization of whole-genome feature of *E. coli* K12 phage A6 and its comparison between *E. coli* K12 phages A1, A2 and A7. Phage A6 is represented by the outermost ring (with genome features annotated), followed by first inner ring represented by phage A1, second inner ring represented by phage A2 and the third inner ring represented by phage A7. **B.** Visualization of whole-genome feature of *P. putida* KT2440 phage A8 and its comparison between *P. putida* KT2440 phages A9, A10 and A11. Phage A8 is represented by the outermost ring (with genome features annotated), followed by first inner ring represented by phage A9, second inner ring represented by phage A10 and the third inner ring represented by phage A11.


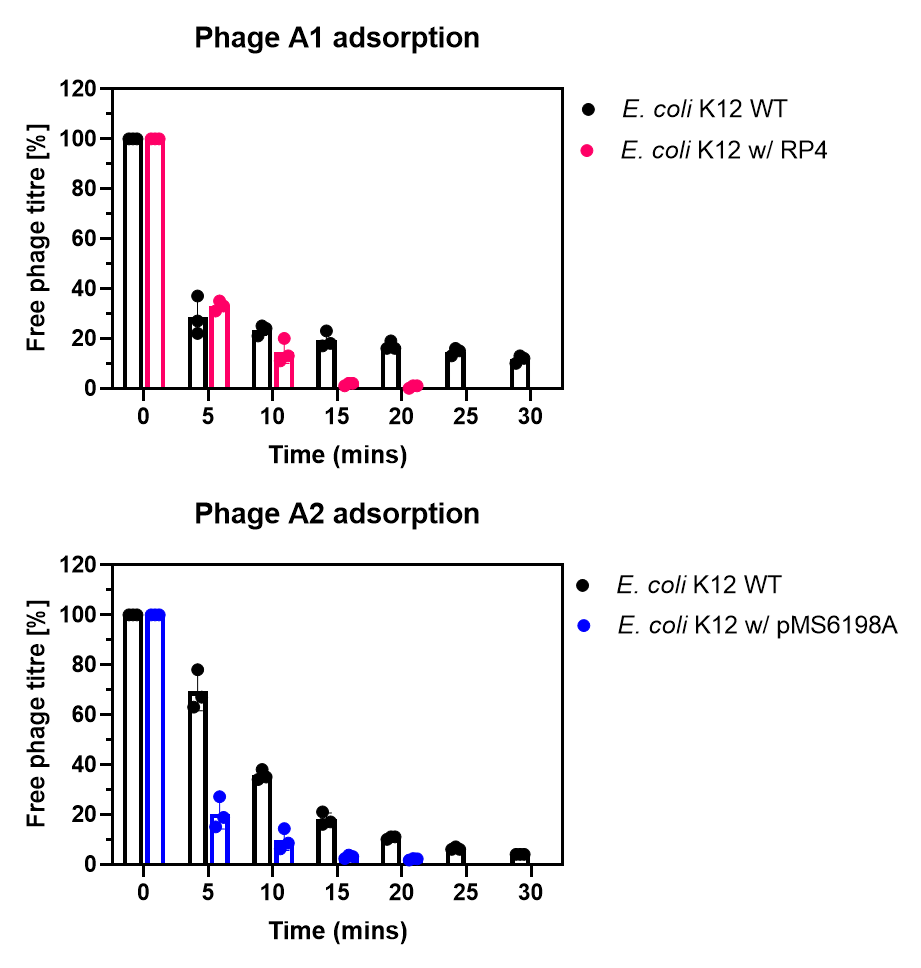


**A**

**B**

**Figure S14**. Expanded phage adsorption of (A) *E. coli* K12 phage A1 and (B) *E. coli* K12 phage A2 against original host (bacteria host for phage isolation) as well as *E. coli* K12 wild type bacteria. Each value is presented as mean values ± standard deviation from three independent experiments. The time point data following maximum phage adsorption were not recorded in the graphs as this corresponds to increase phage titre as a result of phage propagation release after adsorption.


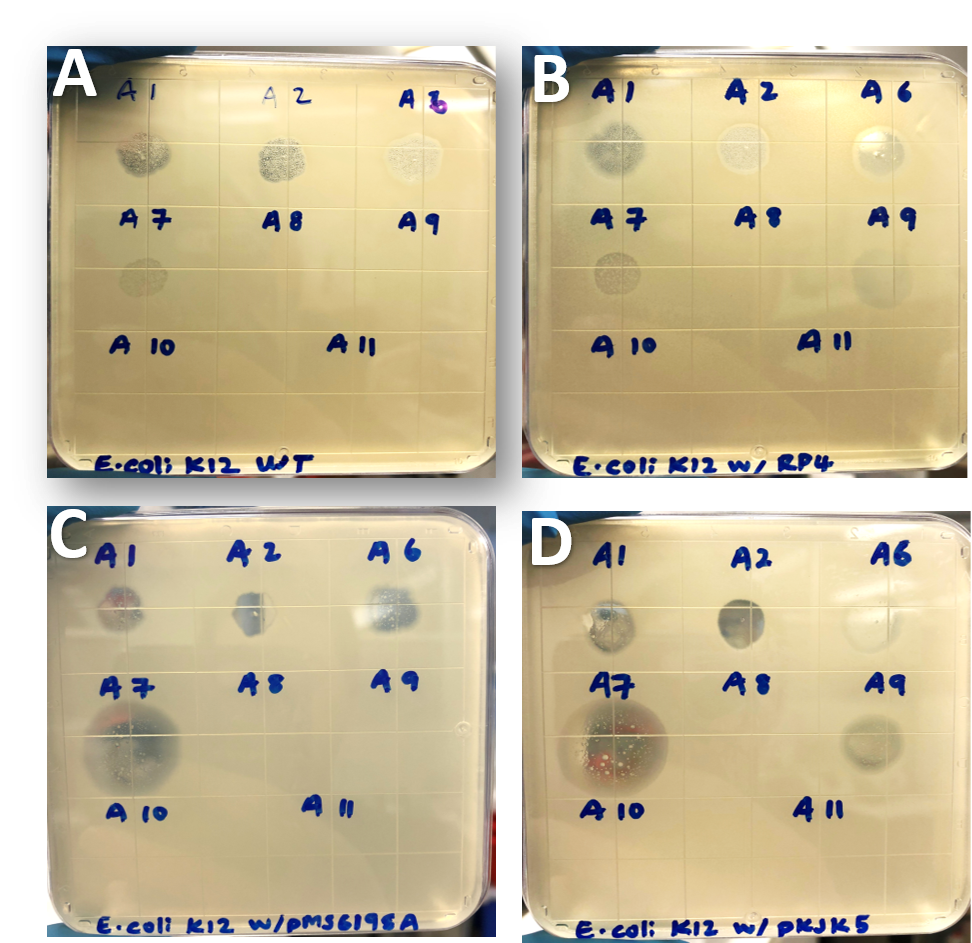


**Figure S15**. Spot test assay of isolated *E. coli* K12 phages (phage A1, A2, A6 and A7) and *P. putida* KT2440 phages (phage A8, A9, A10 and A11) against (A) wild type *E. coli* K12, (B) *E. coli* K12 harbouring plasmid RP4, (C) *E. coli* K12 harbouring plasmid pMS6198A and (D) *E. coli* K12 harbouring plasmid pKJK5.


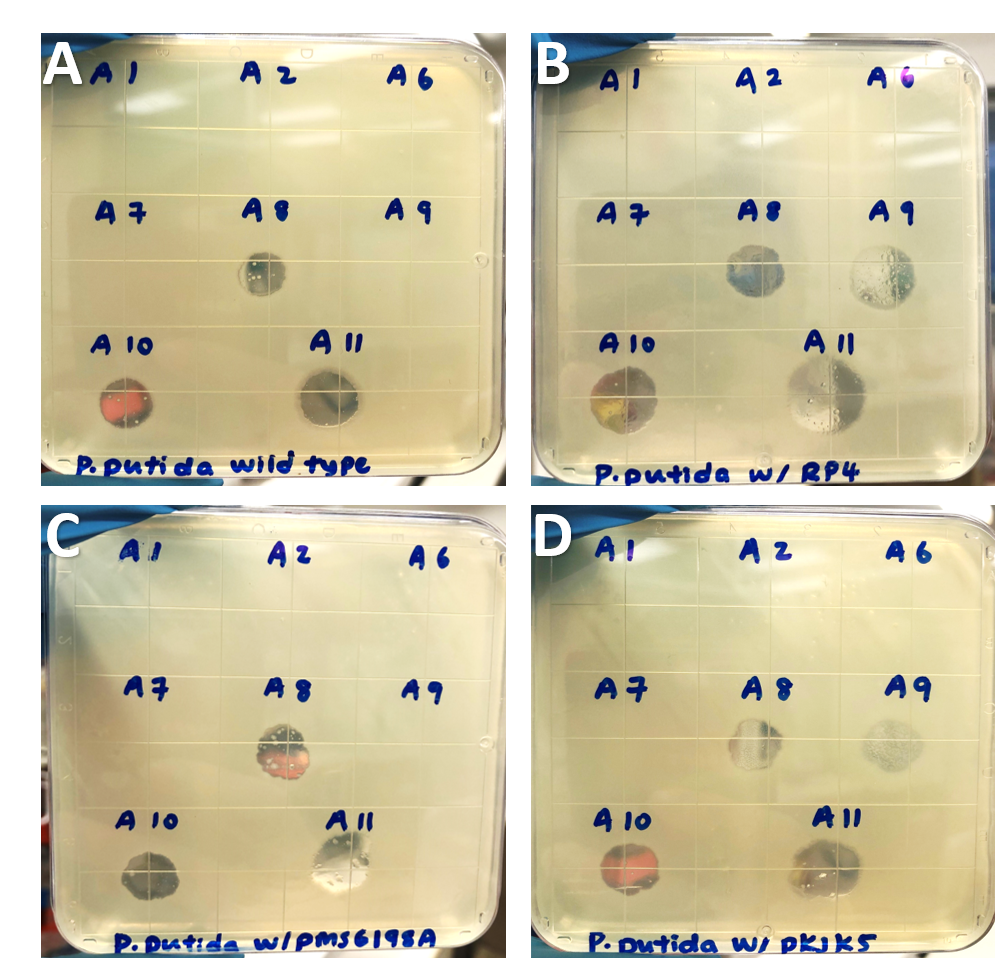


**Figure S16**. Spot test assay of isolated *E. coli* K12 phages (phage A1, A2, A6 and A7) and *P. putida* KT2440 phages (phage A8, A9, A10 and A11) against (A) wild type *P. putida* KT2440 bacteria, (B) *P. putida* KT2440 harbouring plasmid RP4, (C) *P. putida* KT2440 harbouring plasmid pMS6198A and (D) *P. putida* KT2440 harbouring plasmid pKJK5.

**References**

Bahl, M. I., Hansen, L. H., Goesmann, A., & Sørensen, S. J. (2007). The multiple antibiotic resistance IncP-1 plasmid pKJK5 isolated from a soil environment is phylogenetically divergent from members of the previously established α, β and δ sub-groups. *Plasmid, 58*(1), 31-43. doi:10.1016/j.plasmid.2006.11.007

Bolger, A. M., Lohse, M., & Usadel, B. (2014). Trimmomatic: a flexible trimmer for Illumina sequence data. *Bioinformatics, 30*(15), 2114-2120. doi:10.1093/bioinformatics/btu170

Grobbler, C., Virdis, B., Nouwens, A., Harnisch, F., Rabaey, K., & Bond, P. L. (2015). Use of SWATH mass spectrometry for quantitative proteomic investigation of Shewanella oneidensis MR-1 biofilms grown on graphite cloth electrodes. *Systematic and Applied Microbiology, 38*(2), 135-139. doi:10.1016/j.syapm.2014.11.007

Hancock, S. J., Phan, M.-D., Peters, K. M., Forde, B. M., Chong, T. M., Yin, W.-F., . . . Schembri, M. A. (2017). Identification of IncA/C Plasmid Replication and Maintenance Genes and Development of a Plasmid Multilocus Sequence Typing Scheme. *Antimicrobial Agents and Chemotherapy, 61*(2), AAC.01740-01716. doi:10.1128/aac.01740-16

Pansegrau, W., Lanka, E., Barth, P. T., Figurski, D. H., Guiney, D. G., Haas, D., . . . Thomas, C. M. (1994). Complete Nucleotide Sequence of Birmingham IncPα Plasmids. *Journal of Molecular Biology, 239*(5), 623-663. doi:10.1006/jmbi.1994.1404

Wang, Y., Lu, J., Mao, L., Li, J., Yuan, Z., Bond, P. L., & Guo, J. (2019). Antiepileptic drug carbamazepine promotes horizontal transfer of plasmid-borne multi-antibiotic resistance genes within and across bacterial genera. *The ISME Journal, 13*(2), 509-522. doi:10.1038/s41396-018-0275-x

Wick, R. R., Judd, L. M., & Holt, K. E. (2019). Performance of neural network basecalling tools for Oxford Nanopore sequencing. *Genome Biology, 20*(1). doi:10.1186/s13059-019-1727-y
